# Reliability and Validity of Smartphone Cognitive Testing for Frontotemporal Lobar Degeneration

**DOI:** 10.1001/jamanetworkopen.2024.4266

**Published:** 2024-04-01

**Authors:** Adam M. Staffaroni, Annie L. Clark, Jack C. Taylor, Hilary W. Heuer, Mark Sanderson-Cimino, Amy B. Wise, Sreya Dhanam, Yann Cobigo, Amy Wolf, Masood Manoochehri, Leah Forsberg, Carly Mester, Katherine P. Rankin, Brian S. Appleby, Ece Bayram, Andrea Bozoki, David Clark, R. Ryan Darby, Kimiko Domoto-Reilly, Julie A. Fields, Douglas Galasko, Daniel Geschwind, Nupur Ghoshal, Neill Graff-Radford, Murray Grossman, Ging-Yuek Hsiung, Edward D. Huey, David T. Jones, Maria I. Lapid, Irene Litvan, Joseph C. Masdeu, Lauren Massimo, Mario F. Mendez, Toji Miyagawa, Belen Pascual, Peter Pressman, Vijay K. Ramanan, Eliana Marisa Ramos, Katya Rascovsky, Erik D. Roberson, M. Carmela Tartaglia, Bonnie Wong, Bruce L. Miller, John Kornak, Walter Kremers, Jason Hassenstab, Joel H. Kramer, Bradley F. Boeve, Howard J. Rosen, Adam L. Boxer

**Affiliations:** 1 Department of Neurology, Memory and Aging Center, Weill Institute for Neurosciences, University of California, San Francisco; 2 Department of Neurology, Columbia University, New York, New York; 3 Department of Neurology, Mayo Clinic, Rochester, Minnesota; 4 Department of Quantitative Health Sciences, Division of Biomedical Statistics and Informatics, Mayo Clinic, Rochester, Minnesota; 5 Department of Neurology, Case Western Reserve University, Cleveland, Ohio; 6 Department of Neurosciences, University of California, San Diego, La Jolla; 7 Department of Radiology, University of North Carolina, Chapel Hill; 8 Department of Neurology, Indiana University, Indianapolis; 9 Department of Neurology, Vanderbilt University, Nashville, Tennessee; 10 Department of Neurology, University of Washington, Seattle; 11 Department of Psychiatry and Psychology, Mayo Clinic, Rochester, Minnesota; 12 Department of Neurology, Institute for Precision Health, University of California, Los Angeles; 13 Department of Neurology, Knight Alzheimer Disease Research Center, Washington University, Saint Louis, Missouri; 14 Department of Psychiatry, Knight Alzheimer Disease Research Center, Washington University, Saint Louis, Missouri; 15 Department of Neuroscience, Mayo Clinic, Jacksonville, Florida; 16 Department of Neurology, University of Pennsylvania Perelman School of Medicine, Philadelphia; 17 Division of Neurology, University of British Columbia, Musqueam, Squamish & Tsleil-Waututh Traditional Territory, Vancouver, Canada; 18 Department of Neurosciences, University of California, San Diego, La Jolla; 19 Department of Neurology, Nantz National Alzheimer Center, Houston Methodist and Weill Cornell Medicine, Houston Methodist, Houston, Texas; 20 Department of Neurology, UCLA (University of California, Los Angeles); 21 Department of Neurology, University of Colorado, Aurora; 22 Department of Neurology, David Geffen School of Medicine, UCLA; 23 Department of Neurology, University of Alabama, Birmingham; 24 Tanz Centre for Research in Neurodegenerative Diseases, Division of Neurology, University of Toronto, Toronto, Ontario, Canada; 25 Department of Neurology, Massachusetts General Hospital and Harvard Medical School, Boston; 26 Department of Epidemiology and Biostatistics, University of California, San Francisco; 27 Department of Psychological & Brain Sciences, Washington University, Saint Louis, Missouri

## Abstract

**Importance:**

Frontotemporal lobar degeneration (FTLD) is relatively rare, behavioral and motor symptoms increase travel burden, and standard neuropsychological tests are not sensitive to early-stage disease. Remote smartphone-based cognitive assessments could mitigate these barriers to trial recruitment and success, but no such tools are validated for FTLD.

**Objective:**

To evaluate the reliability and validity of smartphone-based cognitive measures for remote FTLD evaluations.

**Design, Setting, and Participants:**

In this cohort study conducted from January 10, 2019, to July 31, 2023, controls and participants with FTLD performed smartphone application (app)–based executive functioning tasks and an associative memory task 3 times over 2 weeks. Observational research participants were enrolled through 18 centers of a North American FTLD research consortium (ALLFTD) and were asked to complete the tests remotely using their own smartphones. Of 1163 eligible individuals (enrolled in parent studies), 360 were enrolled in the present study; 364 refused and 439 were excluded. Participants were divided into discovery (n = 258) and validation (n = 102) cohorts. Among 329 participants with data available on disease stage, 195 were asymptomatic or had preclinical FTLD (59.3%), 66 had prodromal FTLD (20.1%), and 68 had symptomatic FTLD (20.7%) with a range of clinical syndromes.

**Exposure:**

Participants completed standard in-clinic measures and remotely administered ALLFTD mobile app (app) smartphone tests.

**Main Outcomes and Measures:**

Internal consistency, test-retest reliability, association of smartphone tests with criterion standard clinical measures, and diagnostic accuracy.

**Results:**

In the 360 participants (mean [SD] age, 54.0 [15.4] years; 209 [58.1%] women), smartphone tests showed moderate-to-excellent reliability (intraclass correlation coefficients, 0.77-0.95). Validity was supported by association of smartphones tests with disease severity (*r* range, 0.38-0.59), criterion-standard neuropsychological tests (*r* range, 0.40-0.66), and brain volume (standardized β range, 0.34-0.50). Smartphone tests accurately differentiated individuals with dementia from controls (area under the curve [AUC], 0.93 [95% CI, 0.90-0.96]) and were more sensitive to early symptoms (AUC, 0.82 [95% CI, 0.76-0.88]) than the Montreal Cognitive Assessment (AUC, 0.68 [95% CI, 0.59-0.78]) (*z* of comparison, −2.49 [95% CI, −0.19 to −0.02]; *P* = .01). Reliability and validity findings were highly similar in the discovery and validation cohorts. Preclinical participants who carried pathogenic variants performed significantly worse than noncarrier family controls on 3 app tasks (eg, 2-back β = −0.49 [95% CI, −0.72 to −0.25]; *P* < .001) but not a composite of traditional neuropsychological measures (β = −0.14 [95% CI, −0.42 to 0.14]; *P* = .32).

**Conclusions and Relevance:**

The findings of this cohort study suggest that smartphones could offer a feasible, reliable, valid, and scalable solution for remote evaluations of FTLD and may improve early detection. Smartphone assessments should be considered as a complementary approach to traditional in-person trial designs. Future research should validate these results in diverse populations and evaluate the utility of these tests for longitudinal monitoring.

## Key Points

**Question:**

Can remote cognitive testing via smartphones yield reliable and valid data for frontotemporal lobar degeneration (FTLD)?

**Findings:**

In this cohort study of 360 patients, remotely deployed smartphone cognitive tests showed moderate to excellent reliability comparedwith criterion standard measures (in-person disease severity assessments and neuropsychological tests) and brain volumes. Smartphone tests accurately detected dementia and were more sensitive to the earliest stages of familial FTLD than standard neuropsychological tests.

**Meaning:**

These findings suggest that remotely deployed smartphone-based assessments may be reliable and valid tools for evaluating FTLD and may enhance early detection, supporting the inclusion of digital assessments in clinical trials for neurodegeneration.

## Teaser

This cohort study investigates the reliability and validity of unsupervised remote testing of executive functioning and memory via smartphone among patients with frontotemporal lobe degeneration.

## Introduction

Frontotemporal lobar degeneration (FTLD) is a neurodegenerative pathology causing early-onset dementia syndromes with impaired behavior, cognition, language, and/or motor functioning.^1^ Although over 30 FTLD trials are planned or in progress, there are several barriers to conducting FTLD trials. Clinical trials for neurodegenerative disease are expensive,^2^ and frequent in-person trial visits are burdensome for patients, caregivers, and clinicians,^3^ a concern magnified in FTLD by behavioral and motor impairments. Given the rarity and geographical dispersion of eligible participants, FTLD trials require global recruitment,^4^ particularly for those that are far from expert FTLD clinical trial centers. Furthermore, criterion standard neuropsychological tests are not adequately sensitive until symptoms are already noticeable to families, limiting their usefulness as outcomes in early-stage FTLD treatment trials.^4^

Reliable, valid, and scalable remote data collection methods may help surmount these barriers to FTLD clinical trials. Smartphones are garnering interest across neurological conditions as a method for administering remote cognitive and motor evaluations. Preliminary evidence supports the feasibility, reliability, and/or validity of unsupervised smartphone cognitive and motor testing in older adults at risk for Alzheimer disease,^5,6,7,8^ Parkinson disease,^9^ and Huntington disease.^10^ The clinical heterogeneity of FTLD necessitates a uniquely comprehensive smartphone battery. In the ALLFTD Consortium (Advancing Research and Treatment in Frontotemporal Lobar Degeneration [ARTFLD] and Longitudinal Evaluation of Familial Frontotemporal Dementia Subjects [LEFFTDS]), the ALLFTD mobile Application (ALLFTD-mApp) was designed to remotely monitor cognitive, behavioral, language, and motor functioning in FTLD research. Taylor et al^11^ recently reported that unsupervised ALLFTD-mApp data collection through a multicenter North American FTLD research network was feasible and acceptable to participants. Herein, we extend that work by investigating the reliability and validity of unsupervised remote smartphone tests of executive functioning and memory in a cohort with FTLD that has undergone extensive phenotyping.

## Methods

### Participants

Participants were enrolled from ongoing FTLD studies requiring in-person assessment, including participants from 18 centers from the ALLFTD study study^12^ and University of California, San Francisco (UCSF) FTLD studies. To study the app in older individuals, a small group of older adults without functional impairment was recruited from the UCSF Brain Aging Network for Cognitive Health. All study procedures were approved by the UCSF or Johns Hopkins Central Institutional Review Board. All participants or legally authorized representatives provided written informed consent. The study followed the Strengthening the Reporting of Observational Studies in Epidemiology (STROBE) reporting guideline.

Inclusion criteria were age 18 years or older, having access to a smartphone, and reporting English as the primary language. Race and ethnicity were self reported by participants using options consistent with the National Alzheimer’s Coordinating Center (NACC) Uniform Data Set (UDS) and were collected to contextualize the generalizability of these results. Participants were asked to complete tests on their own smartphones. Informants were encouraged for all participants and required for those with symptomatic FTLD (Clinical Dementia Rating Scale plus NACC FTLD module [CDR plus NACC-FTLD] global score ≥1). Recruitment targeted individuals with CDR plus NACC-FTLD global scores less than 2, but sites had discretion to enroll more severely impaired participants. Exclusion criteria were consistent with the parent ALLFTD study.^12^

### Study Design

Participants were enrolled in the ALLFTD-mApp study within 90 days of annual ALLFTD study visits (including neuropsychological and neuroimaging data collection). Site research coordinators (including J.C.T., A.B.W., S.D., and M.M.) assisted participants with app download, setup, and orientation and observed participants completing the first questionnaire. All cognitive tasks were self-administered without supervision (except pilot participants, discussed below) in a predefined order with minor adjustments throughout the study. Study partners of participants with symptomatic FTLD were asked to remain nearby during participation to help navigate the ALLFTD-mApp but were asked not to assist with testing.

The baseline participation window was divided into three 25- to 35-minute assessment sessions occurring over 11 days. All cognitive tests were repeated in every session to enhance task reliability^6,13^ and enable assessment of test-retest reliability, except for card sort, which was administered once every 6 months due to expected practice effects. Adherence was defined as the percentage of all available tasks that were completed. Participants were asked to complete the triplicate of sessions every 6 months for the duration of the app study. Only the baseline triplicate was analyzed in this study.

Replicability was tested by dividing the sample into a discovery cohort (n = 258) comprising all participants enrolled until the initial data freeze (October 1, 2022) and a validation cohort (n = 102) comprising participants enrolled after October 1, 2022, and 18 pilot participants^11^ who completed the first session in person with an examiner present during cognitive pretesting. Sensitivity analyses excluded this small pilot cohort.

### ALLFTD Mobile App

**Figure 1.**
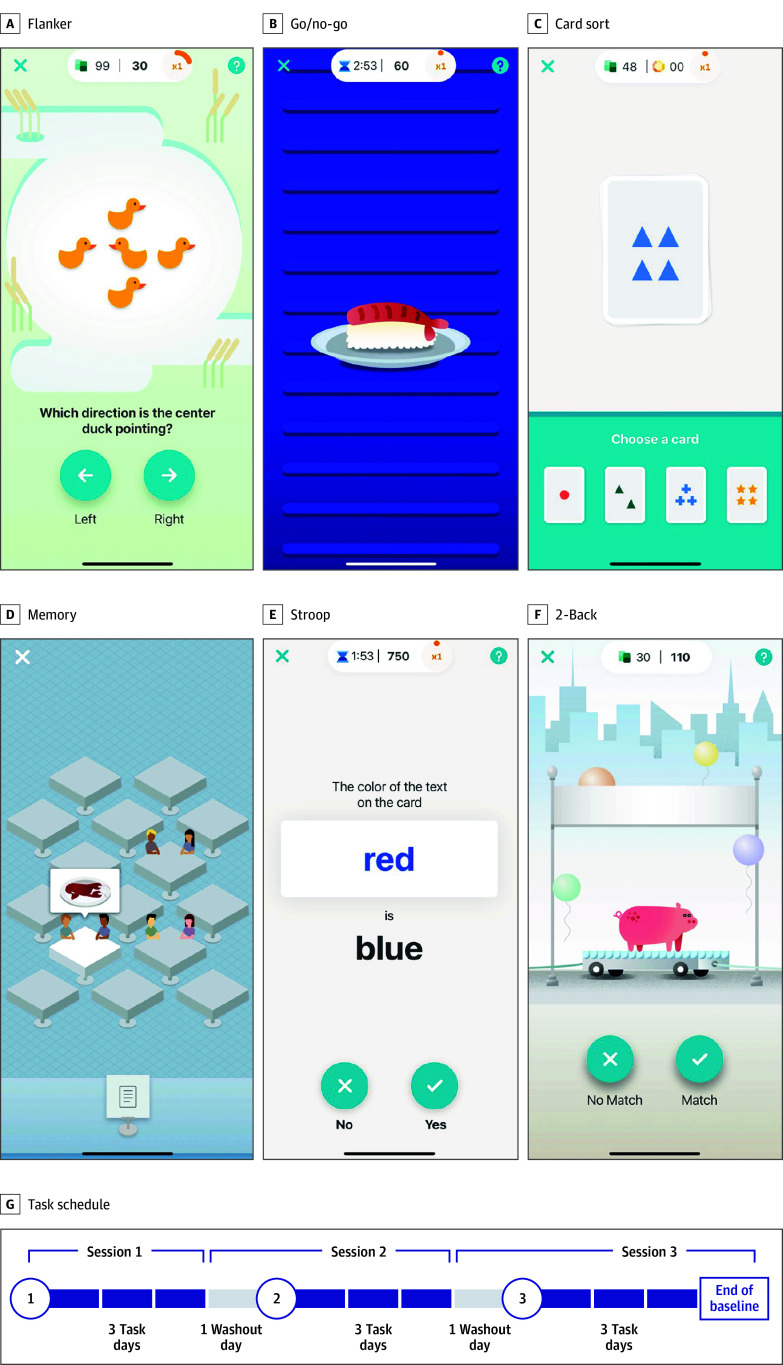
Screenshots of Smartphone Cognitive Tests and Testing Schedule Screenshots of the smartphone cognitive tasks developed by Datacubed Health and included in the ALLFTD Mobile App. Details about the task design and instructions are included in the eMethods in Supplement 1. A, Flanker (Ducks in a Pond) is a task of cognitive control requiring participants to select the direction of the center duck. B, Go/no-go (Go Sushi Go!) requires participants to quickly tap on pieces of sushi (go) but not to tap when they see a fish skeleton (no-go). C, Card sort (Card Shuffle) is a task of cognitive flexibility requiring participants to learn rules that change during the task. D, The adaptative, associative memory task (Humi’s Bistro) requires participants to learn the food orders of several restaurant tables. E, Stroop (Color Clash) is a cognitive inhibition paradigm requiring participants to inhibit their tendency to read words and instead respond based on the color of the word. F, The 2-back task (Animal Parade) requires participants to determine whether animals on a parade float match the animals they saw 2 stimuli previously. G, Participants are asked to complete 3 testing sessions over 2 weeks. Shown in dark blue, they have 3 days to complete each testing session with a washout day between sessions on which no tests are available. Session 2 always begins on day 5 and session 3 on day 9. Screenshots are provided with permission from Datacubed Health.

ALLFTD investigators partnered with Datacubed Health^14^ to develop the ALLFTD-mApp on Datacubed Health’s Linkt platform. The app includes cognitive, motor, and speech tasks. This study focuses on 6 cognitive tests developed by Datacubed Health^11^ comprising an adaptive associative memory task (Humi’s Bistro) and gamified versions of classic executive functioning paradigms: flanker (Ducks in a Pond), Stroop (Color Clash), 2-back (Animal Parade), go/no-go (Go Sushi Go!), and card sort (Card Shuffle) (Figure 1 and eMethods in Supplement 1). Most participants with symptomatic FTLD (49 [72.1%]) were not administered Stroop or 2-back, as pilot studies identified these as too difficult.^11^ The app test results were summarized as a composite score (eMethods in Supplement 1). Participants completed surveys to assess technological familiarity (daily or less than daily use of a smartphone) and distractions (present or absent).

### Clinical Assessment and Traditional Neuropsychological Measures

Criterion standard clinical data were collected during parent project visits. Syndromic diagnoses were made according to published criteria^15,16,17,18,19^ based on multidisciplinary conferences that considered neurological history, neurological examination results, and collateral interview.^20^

The CDR plus NACC-FTLD module is an 8-domain rating scale based on informant and participant report.^21^ A global score was calculated to categorize disease severity as asymptomatic or preclinical if a pathogenic variant carrier (0), prodromal (0.5), or symptomatic (1.0-3.0).^22^ A sum of the 8 domain box scores (CDR plus NACC-FTLD sum of boxes) was also calculated.^22^

Participants completed the UDS Neuropsychological Battery, version 3.0^23^ (eMethods in Supplement 1), which includes traditional neuropsychological measures and the Montreal Cognitive Assessment (MoCA), a global cognitive screen. Executive functioning and processing speed measures were summarized into a composite score (UDS3-EF).^24^ Participants also completed a 9-item list-learning memory test (California Verbal Learning Test, 2nd edition, Short Form).^25^ Most (339 [94.2%]) neuropsychological evaluations were conducted in person. In a subsample (n = 270), motor speed and dexterity were assessed using the Movement Disorder Society Uniform Parkinson Disease Rating Scale^26^ Finger Tapping subscale (0 indicates no deficits [n = 240]).

### Neuroimaging

We acquired T1-weighted brain magnetic resonance imaging for 199 participants. Details of image acquisition, harmonization, preprocessing, and processing are provided in eMethods in Supplement 1 and prior publications.^27^ Briefly, SPM12 (Statistical Parametric Mapping) was used for segmentation^28^ and Large Deformation Diffeomorphic Metric Mapping for generating group templates.^29^ Gray matter volumes were calculated in template space by integrating voxels and dividing by total intracranial volume in 2 regions of interest (ROIs)^30^: a frontoparietal and subcortical ROI and a hippocampal ROI. Voxel-based morphometry was used to test unbiased voxel-wise associations of volume with smartphone tests (eMethods in Supplement 1).^31,32^

### Genetics

Participants in the ALLFTD study underwent genetic testing^33^ at the University of California, Los Angeles. DNA samples were screened using targeted sequencing of a custom panel of genes previously implicated in neurodegenerative diseases, including *GRN* (138945) and *MAPT* (157140). Hexanucleotide repeat expansions in *C9orf72* (614260) were detected using both fluorescent and repeat-primed polymerase chain reaction analysis.^34^

### Statistical Analysis

Statistical analyses were conducted using Stata, version 17.0 (StataCorp LLC), and R, version 4.4.2 (R Project for Statistical Computing). All tests were 2 sided, with a statistical significance threshold of *P* < .05.

Psychometric properties of the smartphone tests were explored using descriptive statistics. Comparisons between CDR plus NACC-FTLD groups (ie, asymptomatic or preclinical, prodromal, and symptomatic) for continuous variables, including demographic characteristics and cognitive task scores (first exposure to each measure), were analyzed by fitting linear regressions. We used χ^2^ difference tests for frequency data (eg, sex and race and ethnicity).

#### Reliability

Internal consistency, which measures reliability within a task, was estimated for participants’ first exposure to each test using Cronbach α (details in eMethods in Supplement 1). Test-retest reliability was estimated using intraclass correlation coefficients for participants who completed a task at least twice; all exposures were included. Reliability estimates are described as poor (<0.500), moderate (0.500-0.749), good (0.750-0.890), and excellent (≥0.900)^35^; these are reporting rules of thumb, and clinical interpretation should consider raw estimates. We calculated 95% CIs via bootstrapping with 1000 samples.

#### Validity

Validity analyses used participants’ first exposure to each test. Linear regressions were fitted in participants without symptoms with age, sex, and educational level as independent variables to understand the unique contribution of each demographic factor to cognitive test scores. Correlations and linear regression between the app-based tasks and disease severity (CDR plus NACC-FTLD sum of boxes score), neuropsychological test scores, and gray matter ROIs were used to investigate construct validity in the full sample. Demographic characteristics were not entered as covariates because the primary goal was to assess associations between app-based measures and criterion standards, rather than understand the incremental predictive value of app measures. To address potential motor confounds, associations with disease severity were evaluated in a subsample without finger dexterity deficits on motor examination (using the Movement Disorder Society Uniform Parkinson Disease Rating Scale Finger Tapping subscale). To complement ROI-based neuroimaging analysis based on a priori hypotheses, we conducted voxel-based morphometry (eMethods in Supplement 1) to uncover other potential neural correlates of test performance.^31,32^ Finally, we evaluated the association of the number of distractions and operating system with reliability and validity, controlling for age and disease severity, which are predictive factors associated with test performance in correlation analyses.

### Group Comparisons

To evaluate the app’s ability to select participants with prodromal or symptomatic FTLD for trial enrollment, we tested discrimination of participants without symptoms from those with prodromal and symptomatic FTLD. To understand the app’s utility for screening early cognitive impairment, we fit receiver operating characteristics curves testing the predictive value of the app composite, UDS3-EF, and MoCA for differentiating participants without symptoms and those with preclinical FTLD from those with prodromal FTLD; areas under the curves (AUC) for the app and MoCA were compared using the DeLong test in participants with results for both predictive factors.

We compared app performance in preclinical participants who carried pathogenic variants with that in noncarrier controls using linear regression adjusted for age (a predictive factor in earlier models). For this analysis, we excluded those younger than 45 years to remove participants likely to be years from symptom onset based on natural history studies.^4^ We analyzed memory performance in participants who carried *MAPT* pathogenic variants, as early executive deficits may be less prominent.^34,36^

## Results

### Participant Characteristics

Of 1163 eligible participants, 360 were enrolled, 439 were excluded, and 364 refused to participate (additional details are provided in the eResults in Supplement 1). Participant characteristics are reported in Table 1 for the full sample. The discovery and validation cohorts did not significantly differ in terms of demographic characteristics, disease severity, or cognition (eTable 1 in Supplement 1). In the full sample, there were 209 women (58.1%) and 151 men (41.9%), and the mean (SD) age was 54.0 (15.4) years (range, 18-89 years). The mean (SD) educational level was 16.5 (2.3) years (range, 12-20 years). Among the 358 participants with racial and ethnic data available, 340 (95.0%) identified as White. For the 18 participants self-identifying as being of other race or ethnicity, the specific group was not provided to protect participant anonymity. Among the 329 participants with available CDR plus NACC-FTLD scores (Table 1), 195 (59.3%) were asymptomatic or preclinical (Global Score, 0), 66 (20.1%) were prodromal (Global score, 0.5), and 68 (20.7%) were symptomatic (global score, 1.0 or 2.0). Of those with available genetic testing results (n = 222), 100 (45.0%) carried a pathogenic familial FTLD pathogenic variant, including 63 of 120 participants without symptoms and with available results. On average, participants completed 78% of available smartphone measures over a mean (SD) of 2.6 (0.6) sessions.

**Table 1.** Participant Characteristics and Test Scores^a^

Characteristic	Participant group				*P* value	Post hoc comparison^b^
	Full sample (N = 360)	Asymptomatic/preclinical (n = 195)	Prodromal (n = 66)	Symptomatic (n = 68)		
Age, mean (SD), y	54.0 (15.4)	47.1 (14.8)	60.0 (13.1)	66.0 (8.3)	<.001	Asx < Pd < Sx
Sex, No. (%)						
Men	151 (41.9)	58 (29.7)	36 (54.5)	42 (61.8)	<.001	Asx > Pd = Sx
Women	209 (58.1)	137 (70.3)	30 (45.5)	26 (38.2)		
Educational level, mean (SD), y	16.5 (2.3) [n = 359]	16.4 (2.3) [n = 195]	16.4 (2.4) [n = 66]	16.8 (2.4) [n = 68]	.51	Asx = Pd = Sx
Race, No. (%)						
White	340 (95.0)	184 (94.8)	64 (98.5)	65 (95.6)	.46	Asx = Pd = Sx
Other^c^	18 (5.0)	10 (5.2)	1 (1.5)	3 (4.4)		
Diagnosis, No. (%)^d^						
ALS	2 (0.6)	0	0	1 (1.5)	NA	NA
Alzheimer disease syndrome	1 (0.3)	0	0	1 (1.5)	NA	NA
bvFTD	44 (12.2)	0	8 (12.1)	36 (52.9)	NA	NA
CBS	10 (2.8)	0	5 (7.6)	5 (7.4)	NA	NA
Clinically without functional impairment	196 (54.4)	186 (95.4)	5 (7.6)	0	NA	NA
FTD/ALS	4 (1.1)	0	2 (3.0)	2 (2.9)	NA	NA
lvPPA	2 (0.6)	0	0	2 (2.9)	NA	NA
MCI	20 (5.6)	0	20 (30.3)	0	NA	NA
MCI behavior	6 (1.7)	0	6 (9.1)	0	NA	NA
Not available	32 (8.9)	6 (3.1)	1 (1.5)	0	NA	NA
nfvPPA	14 (3.9)	0	10 (15.2)	4 (5.9)	NA	NA
Parkinson disease	1 (0.3)	0	1 (1.5)	0	NA	NA
PSP	10 (2.8)	0	4 (6.1)	6 (8.8)	NA	NA
Psychiatric	4 (1.1)	3 (1.5)	0	1 (1.5)	NA	NA
svPPA	14 (3.9)	0	4 (6.1)	10 (14.7)	NA	NA
**Genetic status**						
Carrier of pathogenic variant, No. (%)	100 (27.8)	63 (32.3)	18 (27.3)	8 (11.8)	.001	Asx = Pd > Sx
* C9orf72*	53 (14.7)	32 (16.4)	10 (15.2)	5 (7.4)		
* GRN*	17 (4.7)	11 (5.6)	2 (3.0)	0		
* MAPT*	26 (7.2)	17 (8.7)	6 (9.1)	2 (2.9)		
Other^e^	4 (1.1)	3 (1.5)	0	1 (1.5)		
Noncarrier, No. (%)	122 (33.9)	57 (29.2)	26 (39.4)	35 (51.5)		
Not yet processed, No. (%)	138 (38.3)	75 (38.5)	22 (33.3)	25 (36.8)		
**Conventional cognitive testing**						
CDR plus NACC-FTLD SB score, mean (SD)	1.7 (3.1) [n = 329]	0.0 (0.0) [n = 195]	1.7 (0.9) [n = 66]	6.8 (3.2) [n = 68]	<.001	Asx < Pd < Sx
MoCA score, mean (SD)	25.6 (5.2) [n = 267]	27.8 (2.1) [n = 168]	25.4 (3.8) [n = 48]	18.3 (7.0) [n = 49]	<.001	Asx > Pd > Sx
**ALLFTD mobile-App**						
Cognitive test scores, mean (SD)						
Flanker	7.1 (0.9) [n = 340]	7.5 (0.5) [n = 195]	6.7 (1.0) [n = 62]	6.2 (1.2) [n = 54]	<.001	Asx > Pd > Sx
Go/no-go	70.0 (13.3) [n = 330]	73.7 (4.7) [n = 183]	69.7 (11.6) [n = 61]	59.7 (23.4) [n = 63]	<.001	Asx = Pd > Sx
Card sort	33.0 (8.5) [n = 276]	36.7 (4.0) [n = 142]	31.1 (8.8) [n = 56]	25.5 (10.2) [n = 56]	<.001	Asx > Pd > Sx
Associative Memory	3.9 (1.0) [n = 353]	4.4 (0.9) [n = 192]	3.5 (0.8) [n = 65]	2.9 (0.8) [n =67]	<.001	Asx > Pd > Sx
Stroop	6.0 (1.1) [n = 274]	6.4 (0.8) [n = 189]	5.5 (1.0) [n = 43]	4.2 (1.4) [n = 19]	<.001	Asx > Pd > Sx
2-Back	1.8 (1.0) [n = 267]	2.1 (0.9) [n = 183]	1.3 (1.0) [n = 43]	0.9 (0.8) [n = 19]	<.001	Asx < Pd = Sx
Adherence, mean (SD), %^f^	77.6 (26.0) [n = 357]	76.9 (25.3) [n = 194]	82.5 (24.1) [n = 65]	74.5 (28.0) [n = 67]	.18	Asx > Pd > Sx
Operating system iPhone, No. (%)^g^	260 (72.2)	140 (71.8)	50 (75.8)	51 (75.0)	.77	Asx > Pd > Sx
Distractions present, No. (%)^h^	168 (59.6)	103 (61.3)	26 (53.1)	28 (53.9)	.78	Asx > Pd > Sx
Daily smartphone use, No. (%)^i^	283 (86.8)	172 (95.6)	52 (85.2)	46 (76.7)	<.001	Asx < Pd = Sx

Abbreviations: ALS, amyotrophic lateral sclerosis; bvFTD, behavioral variant frontotemporal dementia; CBS, corticobasal syndrome; CDR plus NACC-FTLD SB, Clinical Dementia Rating Scale plus National Alzheimer’s Coordinating Center Frontotemporal Lobar Degeneration Module Sum of Boxes; lvPPA, logopenic variant primary progressive aphasia; MCI, mild cognitive impairment; MoCA, Montreal Cognitive Assessment; NA, not applicable; nfvPPA, nonfluent variant primary progressive aphasia; PSP, progressive supranuclear palsy; svPPA, semantic variant primary progressive aphasia.

^a^ Full sample includes all participants, including 31 who did not have an available CDR plus NACC FTLD rating. Asymptomatic includes adults without functional impairment and carriers of preclinical pathogenic variants. For continuous variables, number of participants with available data are given. For binary variables, number of participants in the category are given.

^b^ Apply Tukey correction (continuous) or Bonferroni (categorical) for multiple comparisons to compare asymptomatic (Asx; CDR plus NACC FTLD score = 0), prodromal (Pd; CDR plus NACC FTLD score = 0.5), and symptomatic (Sx; CDR plus NACC FTLD score ≥1.0) groups.

^c^ Owing to the small sample size, the specific groups are not provided to protect participant anonymity.

^d^ Diagnostic categories were not compared across groups as diagnoses are designed to differ with increasing disease severity. Note that diagnoses such as psychiatric disorder, Parkinson disease, and Alzheimer disease syndrome are possible manifestations of FTLD; diagnoses of psychiatric disorders were documented in 2 noncarrier controls.

^e^ Refers to identified rare pathogenic variants. The specific variation is not provided to protect participant anonymity.

^f^ Defined as the percentage of all possible tasks that were completed. Eight sessions were removed due to software bugs that prevented participants from completing all tasks.

^g^ Indicates compared with Android.

^h^ Percentage of respondents reporting distractions at baseline of 282 who responded to survey, removing 3 data points reporting at least 8 of 11 distractions. More details are provided in eTables 3 and 4 in Supplement 1.

^i^ Includes those who completed the survey (n = 326).

### Psychometrics

Descriptive statistics for each task are presented in Table 2. Ceiling effects were not observed for any tests. A small percentage of participants were at the floor for flanker (19 [5.3%]), go/no-go (13 [4.0%]), and card sort (9 [3.3%]) scores. Floor effects were only observed in participants with prodromal or symptomatic FTLD.

**Table 2.** Psychometric Properties of the Smartphone Mobile-App Cognitive Tests

Descriptive statistics	App test^a^					
	Flanker	Go/no-go	Card sort	Memory	Stroop	2-Back
**Full sample**						
No. of participants	359	330	276	353	274	267
No. of repeats, mean (SD)	2.6 (0.6)	2.5 (0.7)	NA	2.6 (0.6)	2.6 (0.6)	2.5 (0.7)
Score, mean (SD) [range]	6.8 (1.4) [0.1 to 8.6]	70.0 (13.3) [−1.0 to 83.0]	33.0 (8.5) [8.0 to 43.0]	3.9 (1.0) [1.2 to 6.8]	6.0 (1.1) [1.1 to 2.0]	1.8 (1.0) [−1.0 to 4.2]
Floor, No. (%)	19 (5.3)	13 (4.0)	9 (3.3)	0	0	0
Ceiling, No. (%)	0	0	0	0	0	0
Skewness coefficient	−2.5	−3.3	−1.4	0.0	−1.0	−0.4
Reliability						
Internal consistency, Cronbach α (95% CI)	0.99 (0.99 to 0.99)	0.60 (0.51 to 0.67)	0.92 (0.90 to 0.93)	NA	0.84 (0.81 to 0.87)	0.92 (0.90 to 0.93)
Test-retest, ICC (95% CI)	0.95 (0.93 to 0.96)	0.77 (0.69 to 0.83)	NA	0.79 (0.72 to 0.85)	0.84 (0.78 to 0.88)	0.77 (0.68 to 0.84)
**Participants without symptoms and those with preclinical FTLD **						
No. of participants	195	183	142	192	189	183
No. of repeats, mean (SD)	2.6 (0.6)	2.5 (0.6)	NA	2.5 (0.6)	2.6 (0.6)	2.5 (0.6)
Score, mean (SD) [range]	7.5 (0.5) [5.9 to 8.6]	73.7 (4.7) [57.0 to 82.0]	36.7 (4.0) [16.0 to 43.0]	4.4 (0.9) [1.8 to 6.8]	6.4 (0.8) [4.0 to 8.0]	2.1 (0.9) [−0.2 to 4.2]
Floor, No. (%)	0	0	0	0	0	0
Ceiling, No. (%)	0	0	0	0	0	0
Skewness	−1.0	−0.7	−2.3	0.0	−0.7	−0.1
Reliability						
Internal consistency, Cronbach α (95% CI)	0.99 (0.98 to 0.99)	0.63 (0.52 to 0.72)	0.82 (0.79 to 0.87)	NA	0.84 (0.79 to 0.87)	0.88 (0.84 to 0.90)
Test-retest, ICC (95% CI)	0.85 (0.78 to 0.90)	0 (to 0.34 to 0.15)	NA	0.79 (0.65 to 0.88)	0.80 (0.79 to 0.88)	0.67 (0.52 to 0.77)
**Participants with prodromal and symptomatic FTLD**						
No. of participants	134	124	112	132	62	62
No. of repeats, mean (SD)	2.6 (0.6)	2.5 (0.7)	NA	2.6 (0.7)	2.7 (0.5)	2.5 (0.7)
Score, mean (SD) [range]	6.0 (1.8) [0.1 to 8.0]	64.6 (19.2) [−1.0 to 83.0]	28.3 (9.9) [8.0 to 43.0]	3.2 (0.9) [1.2 to 5.6]	5.1 (1.3) [2.0 to 7.2]	1.2 (0.9) [−0.3 to 3.9]
Floor, No. (%)	18 (13.4)	12 (9.7)	7 (6.3)	0	0	0
Ceiling, No. (%)	0	0	0	0	0	0
Skewness	−1.6	−2.0	−0.5	0.2	−0.5	0.7
Reliability						
Internal consistency, Cronbach α (95% CI)	0.99 (0.98 to 0.99)	0.58 (0.44 to 0.68)	0.91 (0.89 to 0.93)	NA	0.79 (0.69 to 0.85)	0.90 (0.83 to 0.93)
Test-retest, ICC (95% CI)	0.92 (0.88 to 0.95)	0.82 (0.70 to 0.90)	NA	0.77 (0.62 to 0.86)	0.83 (0.69 to 0.91)	0.82 (0.65 to 0.90)

Abbreviations: FTLD, frontotemporal lobar degeneration; ICC, intraclass correlation coefficient; NA, not available.

^a^ Results are presented for the first exposure to each test, except for test-retest estimates, which include data from all available sessions. Number of repeats indicates the mean (SD) number of exposures to each test during participants’ baseline triplicate of sessions (maximum is 3).

### Reliability

**Figure 2.**
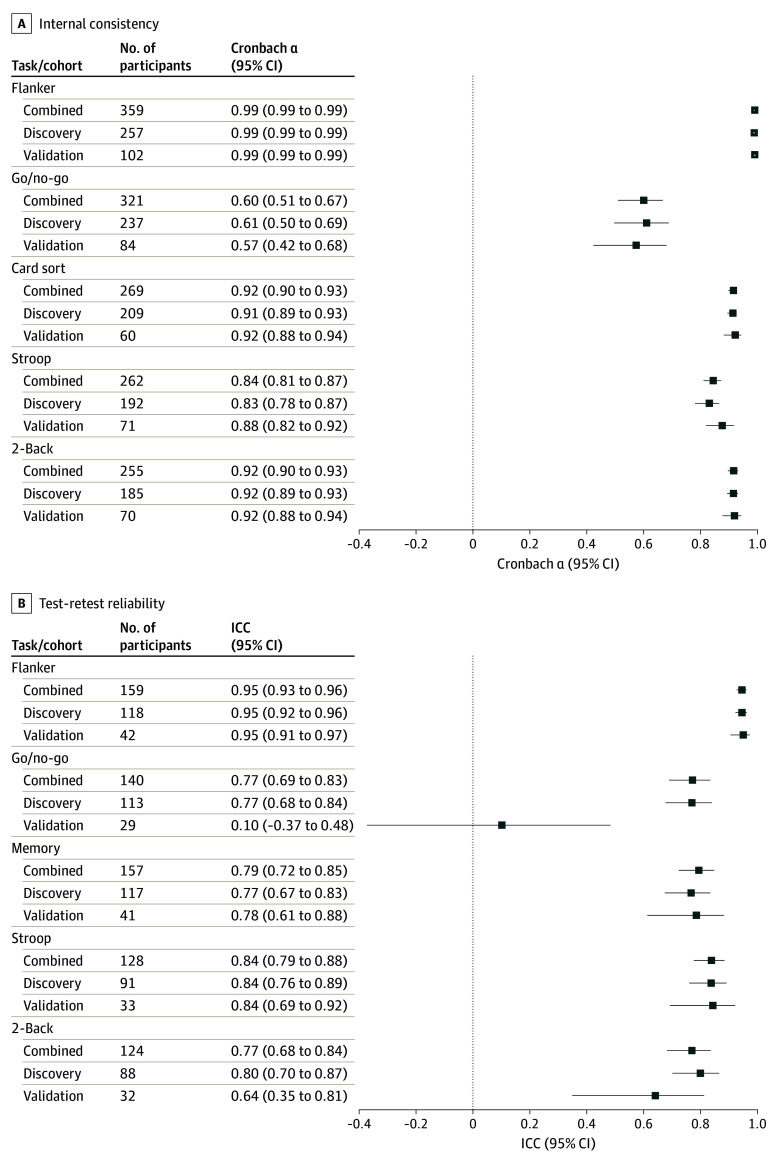
Reliability of Smartphone Cognitive Tests in a Mixed Sample of Adults Without Functional Impairment and Participants With Frontotemporal Lobar Degeneration Forest plots present internal consistency and test-retest reliability results in the discovery and validation cohorts, as well as an estimate in a combined sample of discovery and validation participants. ICC indicates interclass correlation coefficient.

**Figure 3.**
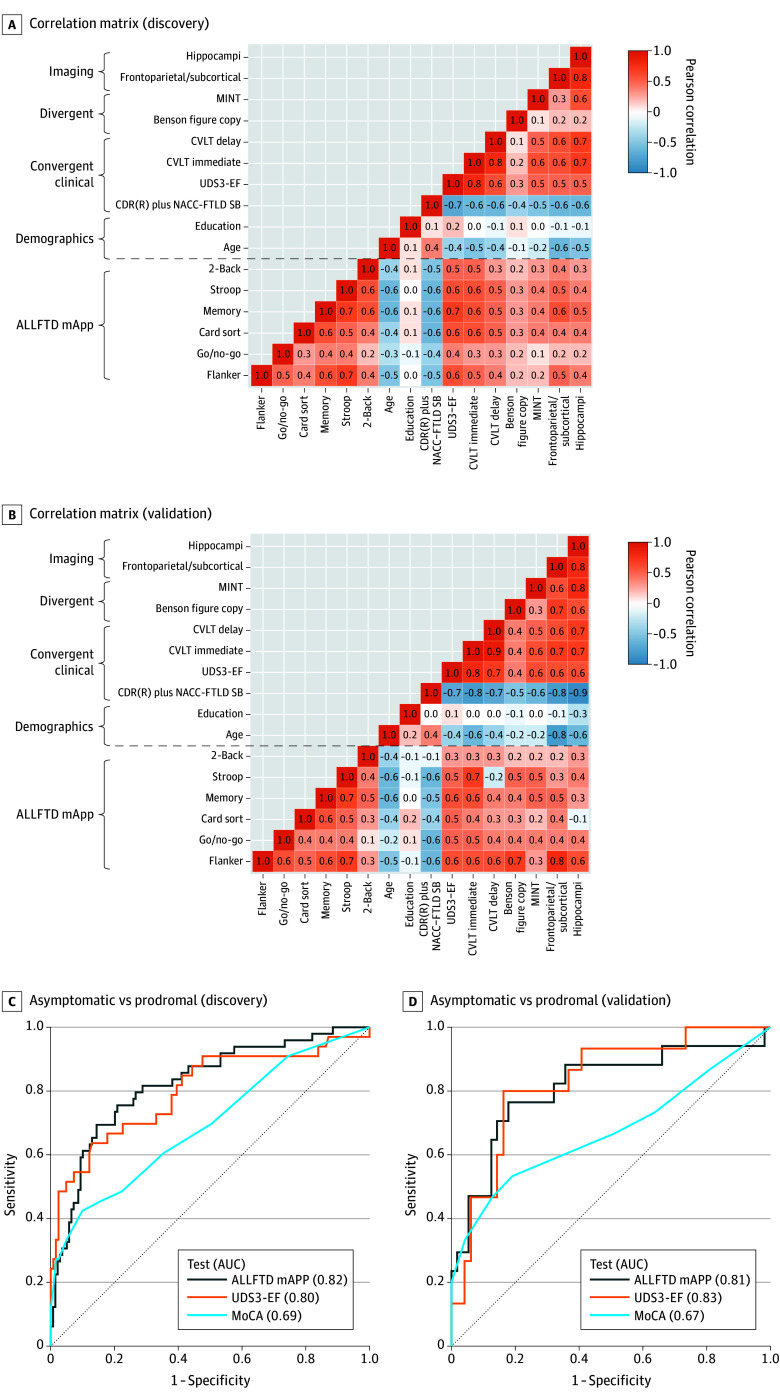
Association of Smartphone Cognitive Tests With Criterion Standards and Detection of Deficits in Early Frontotemporal Lobar Degeneration (FTLD) A and B, Correlation matrices display associations of in-clinic criterion standard measures and ALLFTD mobile App (mApp) test scores in discovery and validation cohorts. Below the horizontal dashed lines, the associations among app tests and between app tests and demographic characteristics convergent clinical measures, divergent cognitive tests, and neuroimaging regions of interest can be viewed. Most app tests show strong correlations with each other and with age, convergent clinical measures, and brain volume. The measures show weaker correlations with divergent measures of visuospatial (Benson Figure Copy) and language (Multilingual Naming Test [MINT]) abilities. The strength of convergent correlations between app measures and outcomes is similar to the correlations between criterion standard neuropsychological scores and these outcomes, which can be viewed by looking across the rows above the horizontal black line. C and D, In the discovery and validation cohorts, receiver operating characteristics curves were calculated to determine how well a composite of app tests, the Uniform Data Set, version 3.0, Executive Functioning Composite (UDS3-EF), and the Montreal Cognitive Assessment (MoCA) discriminate individuals without symptoms (Clinical Dementia Rating Scale plus National Alzheimer’s Coordinating Center FTLD module sum of boxes [CDR plus NACC-FTLD-SB] score = 0) from individuals with the mildest symptoms of FTLD (CDR plus NACC-FTLD-SB score = 0.5). AUC indicates area under the curve; CVLT, California Verbal Learning Test.

Except for go/no-go, internal consistency estimates ranged from good to excellent (Cronbach α range, 0.84 [95% CI, 0.81-0.87] to 0.99 [95% CI, 0.99-0.99]), and test-retest reliabilities were moderate to excellent (interclass correlation coefficient [ICC] range, 0.77 [95% CI, 0.69-0.83] to 0.95 [95% CI, 0.93-0.96]), with slightly higher estimates in participants with prodromal or symptomatic FTLD (Table 2, Figure 2, and eFigure 1 in Supplement 1). Go/no-go reliability was particularly poor in participants without symptoms (ICC, 0.10 [95% CI, −0.37 to 0.48]) and was removed from subsequent validation analyses except the correlation matrix (Figure 3A and B). The 95% CIs for reliability estimates overlapped in the discovery and validation cohorts (Figure 2). Reliability estimates showed overlapping 95% CIs regardless of distractions (eFigure 2 in Supplement 1) or operating systems (eFigure 3 in Supplement 1), with a pattern of slightly lower reliability estimates when distractions were endorsed for all comparisons except Stroop (Cronbach α).

### Construct Validity

In 57 participants without symptoms who did not carry pathogenic variants, older age was associated with worse performance on all measures (β range, *−*0.40 [95 CI, −0.68 to −0.13] to −0.78 [95 CI, −0.89 to −0.52]; *P* ≤ .03), except card sort (β = −0.22 [95% CI, −0.54 to 0.09]; *P* = .16) and go-no/go (β = −0.15 [95% CI, −0.44 to 0.14]; *P* = .31), though associations were in the expected direction. Associations with sex and educational level were not statistically significant.

Cognitive tests administered using the app showed evidence of convergent and divergent validity (eFigure 4 in Supplement 1), with very similar findings in discovery (Figure 3A) and validation cohorts (Figure 3B). App–based measures of executive functioning were generally correlated with criterion standard in-person measures of these domains and less with measures of other cognitive domains (*r* range, 0.40-0.66). For example, the flanker task was associated with the UDS3-EF composite (β = 0.58 [95% CI, 0.48-0.68]; *P* < .001) and measures of visuoconstruction (β for Benson Figure Copy, 0.43 [95% CI, 0.32-0.54]; *P* = .01) and naming (β for Multilingual Naming Test, 0.25 [95% CI, 0.14-0.37]; *P* < .001). The app memory test was associated with criterion standard memory and executive functioning tests.

Worse performance on all app measures was associated with greater disease severity on CDR plus NACC-FTLD (*r* range, 0.38-0.59) (Table 1, Figure 3, and eFigure 4 in Supplement 1). The same pattern of results was observed after excluding those with finger dexterity issues. Except for go/no-go, performance of participants with prodromal FTLD was statistically significantly worse than that of participants without symptoms on all measures (*P* < .001).

The AUC for the app composite to distinguish participants without symptoms from those with dementia was 0.93 (95% CI, 0.90-0.96). The app also accurately differentiated participants without symptoms from those with prodromal or symptomatic FTLD (AUC, 0.87 [95% CI, 0.84-0.92]). Compared with the MoCA (AUC, 0.68 [95% CI, 0.59-0.78), app composite performance (AUC, 0.82 [95% CI, 0.76-0.88]) more accurately differentiated participants without symptoms and with prodromal FTLD (*z* of comparison, −2.49 [95% CI, −0.19 to −0.02]; *P* = .01), with similar accuracy to the UDS3-EF (AUC, 0.81 [95% CI, 0.73-0.88]); highly similar results (eTable 2 in Supplement 1) were observed in the discovery (Figure 3C) and validation (Figure 3D) cohorts.

In 56 participants without symptoms who were older than 45 years, those carrying *GRN*, *C9orf72*, or another rare pathogenic variants performed significantly worse on 3 of 4 executive tests compared with noncarrier controls, including flanker (β = −0.26 [95% CI, −0.46 to −0.05]; *P* = .02), card sort (β = −0.28 [95% CI, −0.54 to −0.30]; *P* = .03), and 2-back (β = −0.49 [95% CI, −0.72 to −0.25]; *P* < .001). The estimated scores of participants who carried pathogenic variants were on average lower than those of carriers on a composite of criterion standard in-person tests, but the difference was not statistically significant (UDS3-EF β = −0.14 [95% CI, −0.42 to 0.14]; *P* = .32). Participants who carried preclinical *MAPT* pathogenic variants scored higher than noncarriers on the app Memory test, though the difference was not statistically significant (β = 0.21 [95% CI, −0.50 to 0.58]; *P* = .19).

In prespecified ROI analyses, worse app executive functioning scores were associated with lower frontoparietal and/or subcortical volume (Figures 3A and B) (β range, 0.34 [95% CI, 0.22-0.46] to 0.50 [95 CI, 0.40-0.60]; *P* < .001 for all) and worse memory scores with smaller hippocampal volume (β = 0.45 [95% CI, 0.34-0.56]; *P* < .001). Voxel-based morphometry (eFigure 5 in Supplement 1) suggested worse app performance was associated with widespread atrophy, particularly in frontotemporal cortices.

### Distractions and Operating System

Only for card sort were distractions (eTables 3 and 4 in Supplement 1) associated with task performance; those experiencing distractions unexpectedly performed better (β = 0.16 [95% CI, 0.05-0.28]; *P* = .005). The iPhone operating system was associated with better performance on 2 speeded tasks: flanker (β = 0.16 [95% CI, 0.07-0.24]; *P* < .001) and go/no-go (β = 0.16 [95% CI, 0.06-0.26]; *P* = .002). In a sensitivity analysis, associations of all app tests with disease severity, UDS3-EF, and regional brain volumes remained after covarying for distractions and operating system, as did the models differentiating participants who carried preclinical pathogenic variants and noncarrier controls.

## Discussion

There is an urgent need to identify reliable and valid digital tools for remote neurobehavioral measurement in neurodegenerative diseases, including FTLD. Prior studies provided preliminary evidence that smartphones collect reliable and valid cognitive data in a variety of age-related and neurodegenerative illnesses. This is the first study, to our knowledge, to provide analogous support for the reliability and validity of remote cognitive testing via smartphones in FTLD and preliminary evidence that this approach improves early detection relative to traditional in-person measures.

Reliability, a prerequisite for a valid clinical trial end point, indicates measurements are consistent. In 2 cohorts, we found smartphone cognitive tests were reliable within a single administration (ie, internally consistent) and across repeated assessments (ie, test-retest reliability) with no apparent differences by operating system. For all measures except go/no-go, reliability estimates were moderate to excellent and on par with other remote digital assessments^5,6,10,37,38^ and in-clinic criterion standards.^39,40,41^ Go/no-go showed similar within- and between-person variability in participants without symptoms (ie, poor reliability), and participant feedback suggested instructions were confusing and the stimuli disappeared too quickly. Those endorsing distractions tended to have lower reliability, though 95% CIs largely overlapped; future research detailing the effect of the home environment on test performance is warranted.

Construct validity was supported by strong associations of smartphone tests with demographics, disease severity, neuroimaging, and criterion standard neuropsychological measures that replicated in a validation sample. These associations were similar to those observed among the criterion standard measures and similar to associations reported in other validation studies of smartphone cognitive tests.^5,6,10^ Associations with disease severity were not explained by motor impairments. The iPhone operating system was associated with better performance on 2 time-based measures, consistent with prior findings.^6^

A composite of brief smartphone tests was accurate in distinguishing dementia from cognitively unimpaired participants, screening out participants without symptoms, and detecting prodromal FTLD with greater sensitivity than the MoCA. Moreover, carriers of preclinical *C9orf72* and *GRN* pathogenic variants performed significantly worse than noncarrier controls on 3 tests, whereas they did not significantly differ on criterion standard measures. These findings are consistent with previous studies showing digital executive functioning paradigms may be more sensitive to early FTLD than traditional measures.^42,43^

### Limitations

This study has some limitations. Validation analyses focused on participants’ initial task exposure. Future studies will explore whether repeated measurements and more sophisticated approaches to composite building (current composite assumes equal weighting of tests) improve reliability and sensitivity, and a normative sample is being collected to better adjust for demographic effects on testing.^24^ Longitudinal analyses will explore whether the floor effects in participants with symptomatic FTLD will affect the utility for monitoring. The generalizability of the findings is limited by the study cohort, which comprised participants who were college educated on average, mostly White, and primarily English speakers who owned smartphones and participated in the referring in-person research study. Equity in access to research is a priority in FTLD research^44,45^; translations of the ALLFTD-mApp are in progress, cultural adaptations are being considered, and devices have been purchased for provisioning to improve the diversity of our sample.

## Conclusions

The findings of this cohort study, coupled with prior reports indicating that smartphone testing is feasible and acceptable to patients with FTLD,^11^ suggest that smartphones may complement traditional in-person research paradigms. More broadly, the scalability, ease of use, reliability, and validity of the ALLFTD-mApp suggest the feasibility and utility of remote digital assessments in dementia clinical trials. Future research should validate these results in diverse populations and evaluate the utility of these tests for longitudinal monitoring.

## Notes

Supplement 1. **eMethods.** Instruments and Statistical Analysis **eResults.** Participants **eTable 1.** Participant Characteristics and Test Scores in Original and Validation Cohorts **eTable 2.** Comparison of Diagnostic Accuracy for ALLFTD Mobile App Composite Score Across Cohorts **eTable 3.** Number of Distractions Reported During the Remote Smartphone Testing Sessions **eTable 4.** Qualitative Description of the Distractions Reported During Remote Testing Sessions **eFigure 1.** Scatterplots of Test-Retest Reliability in a Mixed Sample of Adults Without Functional Impairment and Participants With FTLD **eFigure 2.** Comparison of Test-Retest Reliability Estimates by Endorsement of Distractions **eFigure 3.** Comparison of Test-Retest Reliability Estimates by Operating System **eFigure 4.** Correlation Matrix in the Combined Cohort **eFigure 5.** Neural Correlates of Smartphone Cognitive Test Performance
eReferences

Supplement 2.
Nonauthor Collaborators

Supplement 3.
Data Sharing Statement
