# Supplementary material for: Reliability and Validity of Smartphone Cognitive Testing for Frontotemporal Lobar Degeneration
Source: JAMA Netw Open. 2024 Apr 1;7(4):e244266. doi: 10.1001/jamanetworkopen.2024.4266 (PMC10985553; doi:10.1001/jamanetworkopen.2024.4266)
Supplement: Supplement 1. — eMethods. Instruments and Statistical Analysis eResults. Participants eTable 1. Participant Characteristics and Test Scores in Original and Validation Cohorts eTable 2. Comparison of Diagnostic Accuracy for ALLFTD Mobile App Composite Score Across Cohorts eTable 3. Number of Distractions Reported During the Remote Smartphone Testing Sessions eTable 4. Qualitative Description of the Distractions Reported During Remote Testing Sessions eFigure 1. Scatterplots of Test-Retest Reliability in a Mixed Sample of Adults Without Functional Impairment and Participants With FTLD eFigure 2. Comparison of Test-Retest Reliability Estimates by Endorsement of Distractions eFigure 3. Comparison of Test-Retest Reliability Estimates by Operating System eFigure 4. Correlation Matrix in the Combined Cohort eFigure 5. Neural Correlates of Smartphone Cognitive Test Performance eReferences [file jamanetwopen-e244266-s001.pdf]

Supplementary Online Content

Staffaroni AM, Clark AL, Taylor JC, et al. Reliability and validity of cognitive testing by smartphone in frontotemporal lobar degeneration. *JAMA Netw Open*. 2024;7(4):e244266. doi:10.1001/jamanetworkopen.2024.4266

eMethods. Instruments and Statistical Analysis
eResults. Participants
eTable 1. Participant Characteristics and Test Scores in Original and Validation Cohorts
eTable 2. Comparison of Diagnostic Accuracy for ALLFTD Mobile App Composite Score Across Cohorts
eTable 3. Number of Distractions Reported During the Remote Smartphone Testing Sessions
eTable 4. Qualitative Description of the Distractions Reported During Remote Testing Sessions
eFigure 1. Scatterplots of Test-Retest Reliability in a Mixed Sample of Adults Without Functional Impairment and Participants With FTLD
eFigure 2. Comparison of Test-Retest Reliability Estimates by Endorsement of Distractions
eFigure 3. Comparison of Test-Retest Reliability Estimates by Operating System
eFigure 4. Correlation Matrix in the Combined Cohort
eFigure 5. Neural Correlates of Smartphone Cognitive Test Performance
eReferences

This supplementary material has been provided by the authors to give readers additional information about their work.

© 2024 Staffaroni AM et al. *JAMA Network Open*.

eMethods. Instruments and Statistical Analysis

Uniform Data Set (UDS) Version 3.0 Neuropsychological Tests

Participants were administered the UDS v3.0 neuropsychological battery,[1] which included the Montreal Cognitive Assessment (MoCA), Trail Making Test (TMT) Parts A & B, the Multilingual Naming Test (MINT), Number Span Forward and Backward, Benson Figure Copy and Recall, and Animal and Lexical Fluency.

ALLFTD Mobile App Cognitive Tests

Flanker (*Ducks in a Pond)*

The flanker is a widely used measure of response inhibition and cognitive control.[2–4] In this gamified version, participants are asked to identify the direction (right or left) that a central duck is facing by selecting a button on the screen labeled with either a right- or left-facing arrow. Some trials have four distractor ducks flanking the central duck, all facing either the same (congruent) or opposite (incongruent) direction. The number of congruent (n=45), incongruent (n=45), and non-flanked (n=10) trials are consistent each time a participant takes the task, but the stimuli are presented in a random order each time. Participants provide as many correct responses as possible in 100 trials, with a maximum trial time of 1.5 seconds. An untimed break is provided after the first 50 trials.

The presented internal consistency estimates were conducted on reaction time for incongruent trials. We also analyzed internal consistency of accuracy rather than reaction time, and within congruent trials rather than incongruent trials, and all results were similar (not shown). The primary metric for all other analyses combines accuracy and reaction time using the scoring algorithm that is applied to the flanker task in the NIH Toolbox and EXAMINER.[3,5] The score ranges from 0–10 and enables combining data from more impaired individuals for whom accuracy is more variable, and data from more intact individuals for whom reaction time is more variable. Floor was considered as an accuracy of less than 70% consistent with prior studies;[3] these scores were removed from validation analyses but included in psychometric estimates. Ceiling was defined as a speed/accuracy score of 10.

Go Sushi Go! (Go/No-Go)

In this task, based on the go/no-go paradigm,[6] each trial presents either a piece of sushi (Go trial; 85% of trials) or a fish skeleton (No-Go trial; 15% of trials) on a conveyor belt, and participants are prompted to "grab" the sushi by touching the screen and "ignore" the fish skeleton. In each trial, the stimulus (sushi or skeleton on a plate) is presented for 500 milliseconds and then a lid covers the plate for an additional 500ms until the trial ends. Participants are asked to "grab" the sushi as quickly as possible as soon as it appears on the screen. Participants provide as many correct responses as possible during two 90 second sessions, with an untimed break in between. Stimuli are randomized each time the task is presented.

Presented internal consistency analyses focused on reaction time for No-Go trials; reaction time and accuracy were higher for Go trials (not presented). The primary outcome for all other analyses was total correct minus errors, ignoring trials in which participants did not respond such that timed out trials count against total correct but are not additionally penalized as errors. Floor was defined as less than 40 correct and ceiling as greater than 90 correct based on the sample distribution.

Card Sort (Card Shuffle)

A gamified adaptation of the classic card sorting task, designed to measure set-shifting.[1] Participants must match a card to a target card based on color, shape, or number, but are not provided information about the rules. After 6 consecutive correct answers, the rule changes. The task includes 48 untimed trials that are presented in a random order each time.

Internal consistency analyses used binary accuracy across all trials. The primary outcome for all other analyses was total correct matches. Floor was defined as less than 12 correct (chance) and ceiling as >45/48.

Stroop (Color Clash)

This task is a two-part adaptation of the Stroop test, a classic measure of processing speed and response inhibition.[7] Part one presents a box with either a red, blue, or yellow rectangle and a word in black ink below the box, either "red,"

© 2024 Staffaroni AM et al. *JAMA Network Open*.

"blue," or "yellow." Participants are asked whether the color in the box matches the color word. Participants provide as many correct responses as possible in 30 seconds with no maximum response time (or time-out) for each trial. Part two presents a box with a color word in colored ink and another color word in black ink below the box, and participants are asked whether the word matches the color of the ink. Part two tests cognitive inhibition because the stimulus is now a word that sometimes is incongruent with the ink color. Participants provide as many correct responses as possible in 120 seconds, with a maximum response time of three seconds per trial. Stimuli are randomly presented at each task administration.

Presented internal consistency estimates were conducted on reaction time for incongruent trials. We also analyzed internal consistency of accuracy rather than reaction time, and within congruent trials rather than incongruent trials, and results were grossly similar. The primary outcome for all other analyses was a 0-10 score combining speed and accuracy using the scoring algorithm that is applied to the flanker task in the NIH Toolbox and EXAMINER.[3,5] Accuracy and speed each contribute up to 5 points to the score and enables combining data from more impaired individuals for whom accuracy is more variable, and data from more intact individuals for whom reaction time is more variable. Floor was defined as scores less than 2 and ceiling as a score of 10.

2-Back (Animal Parade)
In this adaptation of the 2-back paradigm,[3] each trial presents an animal on a parade float, and participants are asked to select "Match" when the current animal on the screen is the same as the animal from two trials prior and "No Match" otherwise. Participants complete three blocks of 34 trials with a maximum response time of 1.5 seconds. Animals are presented in a random order each time the task is administered.

Internal consistency analyses used trial-by-trial binary accuracy. The primary metric used for all other analyses is d prime (d'), a signal detection theory metric that incorporates hits and false positives.[3] Floor was defined as less than = -1 and ceiling as a perfect score.

Associative Memory (*Humi's Bistro)*
In this adaptive associative memory task, participants are presented with a restaurant filled with tables. They observe the tables ordering different food items in sequence. Tables are randomly chosen at each task iteration. The participant is asked to learn the food order of each table. Once all orders are displayed, the food items appear at the bottom of the screen and participants are asked to drag and drop each food item to the corresponding table. Participants are allowed seven seconds to deliver each item. Stepwise adaptation is used, such that the participant is first asked to learn the orders of three tables. If they correctly match all tables to food items, the next trial includes one additional table; if only one table had an incorrect or missing plate, the next trial has the same number of tables as the previous trial; if more than one table had an incorrect or missing plate, the next trial would present one less table, never dropping below three tables. Participants are provided feedback about each response. Participants have four minutes to complete as many trials as possible, with an untimed break after two minutes.

Internal consistency analyses used number of correct tables for each trial. The primary outcome for all other analyses is mean number of correct tables. Floor was defined as < 1 average correct tables and ceiling as 12 average correct.

ALLFTD mApp Composite
To minimize multiple comparisons and improve the robustness of the cognitive outcomes used for the logistic regression and voxel-based morphometry analyses, the smartphone tests were combined into a single composite score. For each test, available scores were transformed into sample-based z-scores (mean = 0, standard deviation = 1). For each participant, available z-scores were then averaged to create a composite score. Given the results of the reliability analysis, we excluded Go/No-Go. The logistic regression analysis included asymptomatic and prodromal participants. The available z-scores from 5 tests were then averaged to create a composite for each individual, as long as they had at least 2 of 5 tests.

<u>Reliability</u>

Cronbach's Alpha
Internal consistency was assessed via Cronbach's alphas. Cronbach's alpha is affected by the number of trials completed by an individual, which differed by task (e.g., Stroop vs 2-Back) and comparison group (e.g., Validation

vs Discovery cohort). An unequal number of trials between groups may affect comparisons. As such, we calculated Cronbach's alphas for all possible trials within the full (Combined) sample for each task. We then recorded the number of trials completed by 95% of participants on that task. We retained that number of trials for all Cronbach's alphas within that task across all groups. Analyses were completed within R using the "ltm" package.[8] Confidence intervals were calculated via bootstrapping across 1000 resamples.

Interclass correlation coefficient (ICC)
The ICC for each task was estimated with a one-way analysis of variance (ANOVA) test within R via the "ICC" package.[9] Analyses for each task were restricted to those with at least two timepoints.

Neuroimaging

Image Acquisition
Details of image acquisition, processing, and harmonization have been published elsewhere.[10] ALLFTD participants were scanned at 3T on MRI scanners. T1-weighted images from ALLFTD were acquired as Magnetization Prepared Rapid Gradient Echo (MP-RAGE) images using the following parameters: 240x256x256 matrix; about 170 slices; voxel size = 1.05x1.05x1.25 mm^3; flip angle, TE and TR varied by vendor. A standard imaging protocol was used across all centers, managed and reviewed for quality by a core group at Mayo Clinic, Rochester.

Image Processing
Before any prepossessing of the images, all T1-weighted images were visually inspected for quality control. Images with excessive motion or image artifact were excluded. T1-weighted images underwent bias field correction using N3 algorithm.[11] The segmentation was performed using SPM12 (Wellcome Trust Center for Neuroimaging, London, UK, http://www.fil.ion.ucl.ac.uk/spm) unified segmentation.[12] A customized group template was generated from the segmented gray and white matter tissues and cerebrospinal fluid by non-linear registration template generation using the Large Deformation Diffeomorphic Metric Mapping framework.[13] Subjects' native space gray and white matter were geometrically normalized to the group template, modulated, and then smoothed in the group template. The applied smoothing used a Gaussian kernel with 8~mm full width half maximum. Every step of the transformation was carefully inspected from the native space to the group template.

Regional volume estimates were calculated from individual subjects' smoothed, modulated grey matter in template space, by integrating all voxels in two a priori regions of interest (ROIs)[14]: a frontoparietal and subcortical ROI and a hippocampal ROI. Volume estimates were then represented as percentage of total intracranial volume.

Voxel-based Morphometry (VBM) Methods
VBM is a statistical method for analyzing structural magnetic resonance imaging (MRI) scans. Gray matter atrophy at the voxel level can be compared between two groups, or in this case, correlated with a covariate of interest.[15] VBM was conducted using FSL Randomise with 5,000 permutations and threshold-free cluster enhancement (default parameters: height parameter = 2; E extent parameter = 0.5; C connectivity = 6; p-value < 0.05) to address multiple comparisons.[16] Results within each statistically significant voxel (P < .05 after family-wise error correction) are presented in eFigure 5 as T-statistics, which describe the strength of the association between gray matter volume in that voxel and performance on a composite of smartphone measures.

© 2024 Staffaroni AM et al. *JAMA Network Open*.

eResults

Participants

Of 1163 eligible participants from ALLFTD and other UCSF studies, 360 were enrolled and 364 refused to participate, and 439 were excluded from enrollment into the study due to: 1) CDR®+NACC-FTLD scores >1 (n=73), 2) technical issues (n=10), 3) rescinded consent (n=2), 5) English was not participant's primary language (n = 4), 6) Site staff were unavailable or not yet trained (n=92), 7) participant was non-responsive after consent (n=24), or 8) participant did not complete any tasks (n=83). Note that some participants were excluded for multiple reasons. The reason for exclusion was not clearly documented by the site coordinator for 168 participants.

© 2024 Staffaroni AM et al. *JAMA Network Open*.

eTable 1. Participant Characteristics and Test Scores in Original and Validation Cohorts

	Combined[a]		Discovery		Validation			
	n[b]	Mean (SD)	n	Mean (SD)	n	Mean (SD)	P	Group Comparisons
**A. General Sample Characteristics**								
**Age, y**	360	54.0 (15.4)	258	53.6 (14.7)	102	54.9 (17.2)	0.48	Disc = Val
**Sex, n(%)**								Disc = Val
Male	151	41.9%	105	40.7%	46	45.1%	0.45	Disc = Val
Female	209	58.1%	153	59.3%	56	54.9%		
**Education, y**	359	16.5 (2.3)	257	16.5 (2.2)	102	16.5 (2.6)	0.88	Disc = Val
**Race, n(%)**								
White	340	95.0%	245	95.3%	95	94.1%	0.62	Disc = Val
Other [c]	18	5.0%	12	4.7%	6	5.9%		
**Diagnosis, n(%)** [d]								
ALS	2	0.6%	1	0.40%	1	1.00%		--
Alzheimer's disease dementia	1	0.3%	0	0.00%	1	1.00%		--
bvFTD	44	12.2%	30	11.60%	14	13.70%		--
CBS	10	2.8%	9	3.50%	1	1.00%		--
Clinically normal	196	54.4%	141	54.70%	55	53.90%		--
FTD/ALS	4	1.1%	3	1.20%	1	1.00%		--
lvPPA	2	0.6%	1	0.40%	1	1.00%		--
MCI	20	5.6%	15	5.80%	5	4.90%		--
MCI - behavior	6	1.7%	4	1.60%	2	2.00%		--
NA	32	8.9%	18	7.00%	14	13.70%		--
nfvPPA	14	3.9%	11	4.30%	3	2.90%		--
Parkinson's disease	1	0.3%	1	0.40%	0	0.00%		--
PSP	10	2.8%	9	3.50%	1	1.00%		--
Psychiatric	4	1.1%	4	1.60%	0	0.00%		--
svPPA	14	3.9%	11	4.30%	3	2.90%		
**CDR®+NACC-FTLD SB**	329	1.7 (3.1)	236	1.7 (2.9)	93	1.8 (3.4)	0.70	Disc = Val
**MoCA**	267	25.6 (5.2)	188	25.7 (5.3)	79	25.4 (5.0)	0.65	Disc = Val
**B. ALLFTD Mobile App**								
**Cognitive Test Performance**								
Flanker	340	7.1 (0.9)	240	7.1 (0.8)	100	7.0 (1.2)	0.09	Disc = Val
Go/No-Go	330	70.0 (13.3)	245	69.8 (12.9)	85	70.7 (14.7)	0.59	Disc = Val
Card Sort	276	33.0 (8.5)	216	33.0 (8.3)	60	32.6 (9.3)	0.73	Disc = Val

© 2024 Staffaroni AM et al. *JAMA Network Open*.

Associative Memory	353	3.9 (1.0)	255	3.9 (1.0)	98	3.9 (1.1)	0.95	Disc = Val
Stroop	274	6.0 (1.1)	201	6.1 (1.1)	73	6.0 (1.1)	0.44	Disc = Val
2-Back	267	1.8 (1.0)	195	1.8 (1.0)	72	1.9 (0.9)	0.53	Disc = Val
**Adherence** [e]	357	77.6% (26.0)	256	78.7% (25.2)	101	75.1% (28.0)	0.24	Disc = Val
**Operating System, n (%)** [f]								
iPhone (%)	260	72.2%	193	74.8%	67	65.7%	0.08	Disc = Val
**Distractions (% yes)**	168	59.6%	133	63.9%	35	47.3%	0.11	Disc = Val
**Smartphone Use, n (%)**								
Daily users [g]	283	86.8%	211	84.4%	72	94.7%	0.02	Disc < Val

Abbreviations: ALS, amyotrophic lateral sclerosis; bvFTD, behavioral variant frontotemporal dementia; CBS, corticobasal syndrome; lvPPA, logopenic variant primary progressive aphasia; MCI, mild cognitive impairment; NA, diagnosis unavailable; nfvPPA, nonfluent variant primary progressive aphasia; PSP, progressive supranuclear palsy; svPPA, semantic variant primary progressive aphasia; CDR®+NACC-FTLD SB, Clinical Dementia Rating Scale plus National Alzheimer's Coordinating Center Frontotemporal Lobar Degeneration Module Sum of Boxes; MoCA, Montreal Cognitive Assessment

[a] Combined sample includes all participants from discovery and validation cohorts.
[b] For continuous variables, n = number of participants with available data. For binary variables, n = number of participants in the category.
[c] Other refers to race groups other than White. The specific group is not provided to protect participant anonymity.
[d] Diagnostic categories were not compared across groups as sample sizes are relatively small in validation sample and there is a greater amount of missing data.
[e] Adherence was defined as the percentage of all possible tasks that were completed.
[f] Percentage using iOS compared to Android
[g] Percentages of those who completed the survey (n=326)

© 2024 Staffaroni AM et al. *JAMA Network Open*.

eTable 2. Comparison of Diagnostic Accuracy for ALLFTD Mobile App Composite Score Across Cohorts

CDR® + NACC FTLD Comparison	Cohort	AUC (95%CI)
Asymptomatic (0) vs Symptomatic (≤1)	Combined	0.93 (0.91 to 0.96)
	Discovery	0.94 (0.91 to 0.97)
	Validation	0.90 (0.82 to 0.99)
Asymptomatic (0) vs Prodromal/Dementia (≤0.5)	Combined	0.87 (0.84 to 0.92)
	Discovery	0.88 (0.84 to 0.93)
	Validation	0.86 (0.78 to 0.94)
Asymptomatic (0) vs Prodromal (0.5)	Combined	0.82 (0.76 to 0.88)
	Discovery	0.82 (0.75 to 0.89)
	Validation	0.81 (0.68 to 0.95)

© 2024 Staffaroni AM et al. *JAMA Network Open*.

eTable 3. Number of Distractions Reported During the Remote Smartphone Testing Sessions

Session	Number Endorsed Distractions	Average Number of Distractions
1	139/267	1.54
2	93/229	1.62
3	67/160	1.52

Note: This table displays the number of individuals who endorsed one or more distractions by smartphone testing session. For those who endorsed distractions, the average number of distractions endorsed is displayed.

© 2024 Staffaroni AM et al. *JAMA Network Open*.

eTable 4. Qualitative Description of the Distractions Reported During Remote Testing Sessions

**Distraction Type Endorsed**	**Session 1**, N = 267	**Session 2**, N = 229	**Session 3**, N = 160
None	47% (126)	59% (134)	58% (93)
Television	6.4% (17)	3.9% (9)	1.3% (2)
Radio/music	2.2% (6)	0.9% (2)	1.9% (3)
Voices/conversations	15% (41)	11% (26)	8.9% (14)
Phone notifications	9.7% (26)	7.4% (17)	9.6% (15)
Computer distractions (notifications/emails/videos/ads/etc.)	2.2% (6)	1.7% (4)	1.3% (2)
Outside noises	11% (30)	9.2% (21)	7.6% (12)
Air conditioning	9.7% (26)	6.1% (14)	6.4% (10)
House sounds (cooking/cleaning/etc.)	4.9% (13)	6.6% (15)	11% (18)
Family or pets	12% (33)	11% (26)	14% (22)
Technical Issue	0.7% (2)	0.4% (1)	0.6% (1)
Internal distractor (headache/difficulty focusing/fatigue/etc.)	2.2% (6)	0.9% (2)	1.9% (3)

Note: This table displays the percent of participants (and number of participants) who endorsed each type of distraction stratified by App session. The total number of participants who completed a distraction survey in each chapter is included at the top of the column. As participants were able to endorse multiple distractions in their response, total percent and total N for each column do not add up to 100%. Participants who endorsed “other” distractions (n=84) were asked to provide further details using free text response; those responses were categorized into the closest matching category.

© 2024 Staffaroni AM et al. *JAMA Network Open*.

eFigure 1. Scatterplots of Test-Retest Reliability in a Mixed Sample of Adults Without Functional Impairment and Participants With FTLD

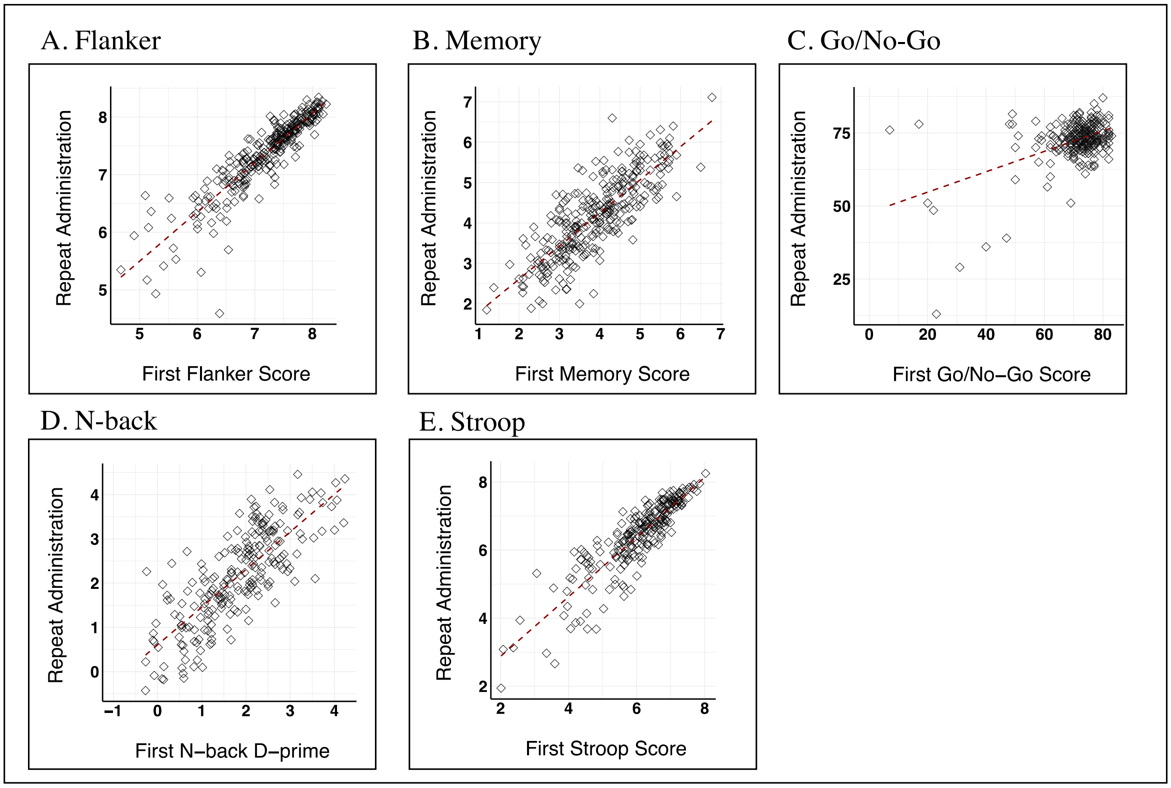

Note. For each smartphone task, participants' first exposure was plotted against the average of the available follow-up testing sessions. All measures showed moderate-to-excellent test-retest reliability with the exception of Go/No-Go, which showed poor convergence between first and repeat administrations, which is visible in Panel C. Flanker scores <4 were removed for plotting purposes (n=20).

© 2024 Staffaroni AM et al. *JAMA Network Open*.

eFigure 2. Comparison of Test-Retest Reliability Estimates by Endorsement of Distractions

2A. Internal Consistency

Task	Cohort	N	Alpha	95% CI
Flanker	Distraction -	184	0.99	(0.99 to 0.99)
	Distraction +	168	0.99	(0.98 to 0.99)
Go/No-Go	Distraction -	180	0.63	(0.52 to 0.71)
	Distraction +	162	0.6	(0.46 to 0.69)
Card Sort	Distraction -	125	0.92	(0.90 to 0.93)
	Distraction +	83	0.91	(0.87 to 0.93)
Stroop	Distraction -	133	0.82	(0.76 to 0.87)
	Distraction +	133	0.86	(0.81 to 0.90)
2-Back	Distraction -	137	0.93	(0.91 to 0.94)
	Distraction +	131	0.9	(0.87 to 0.92)

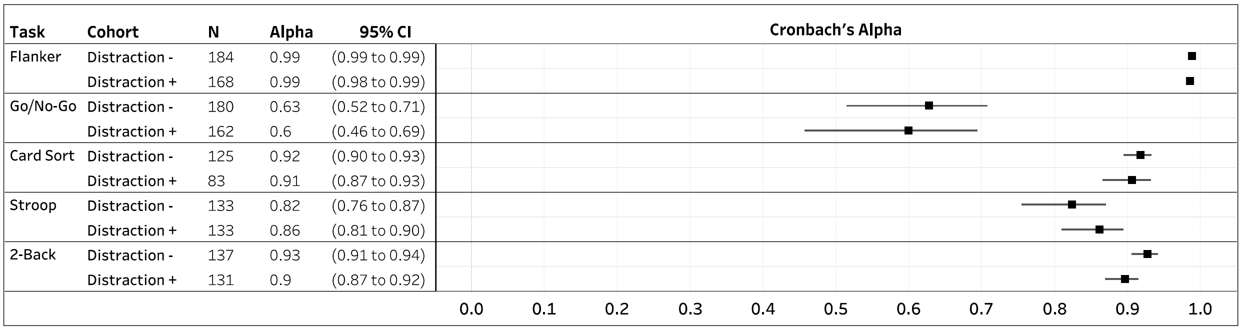

2B. Test-Retest Reliability

Task	Cohort	N	ICC	95% CI
Flanker	Distraction -	71	0.91	(0.85 to 0.95)
	Distraction +	54	0.91	(0.84 to 0.95)
Go/No-Go	Distraction -	71	0.74	(0.58 to 0.84)
	Distraction +	54	0.67	(0.48 to 0.80)
Memory	Distraction -	71	0.83	(0.73 to 0.90)
	Distraction +	54	0.79	(0.65 to 0.88)
Stroop	Distraction -	57	0.89	(0.81 to 0.94)
	Distraction +	41	0.83	(0.68 to 0.91)
2-Back	Distraction -	57	0.87	(0.78 to 0.93)
	Distraction +	41	0.76	(0.56 to 0.87)

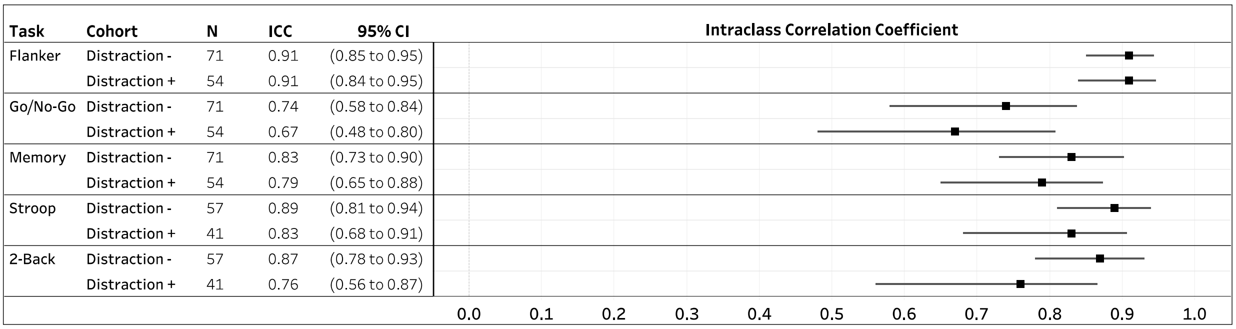

© 2024 Staffaroni AM et al. *JAMA Network Open*.

eFigure 3. Comparison of Test-Retest Reliability Estimates by Operating System

3A. Internal Consistency

Task	Cohort	N	Alpha	95% CI
Flanker	Android	100	0.99	(0.99 to 0.99)
	iOS	259	0.99	(0.99 to 0.99)
Go/No-Go	Android	92	0.66	(0.54 to 0.75)
	iOS	229	0.57	(0.44 to 0.66)
Card Sort	Android	77	0.93	(0.90 to 0.94)
	iOS	192	0.91	(0.89 to 0.93)
Stroop	Android	74	0.86	(0.78 to 0.90)
	iOS	188	0.84	(0.79 to 0.87)
2-Back	Android	73	0.9	(0.87 to 0.92)
	iOS	182	0.92	(0.90 to 0.94)

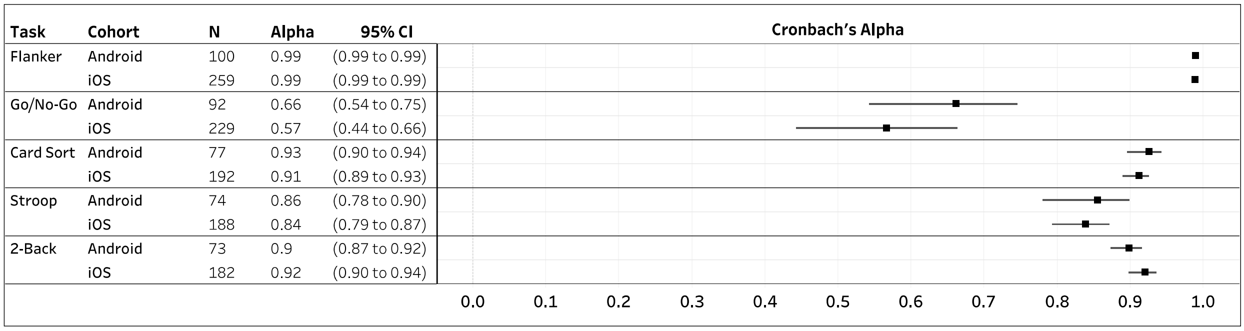

3B. Test-Retest Reliability

Task	Cohort	N	ICC	95% CI
Flanker	Android	44	0.97	(0.95 to 0.99)
	iOS	115	0.94	(0.91 to 0.96)
Go/No-Go	Android	38	0.85	(0.71 to 0.92)
	iOS	101	0.73	(0.61 to 0.81)
Memory	Android	41	0.84	(0.70 to 0.91)
	iOS	115	0.78	(0.69 to 0.84)
Stroop	Android	38	0.77	(0.58 to 0.87)
	iOS	90	0.86	(0.79 to 0.90)
2-Back	Android	35	0.86	(0.72 to 0.93)
	iOS	88	0.72	(0.59 to 0.81)

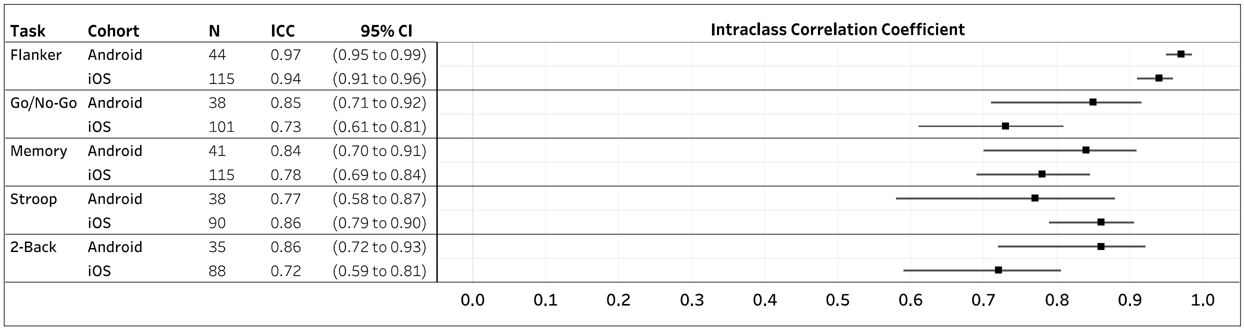

© 2024 Staffaroni AM et al. *JAMA Network Open*.

eFigure 4. Correlation Matrix in the Combined Cohort

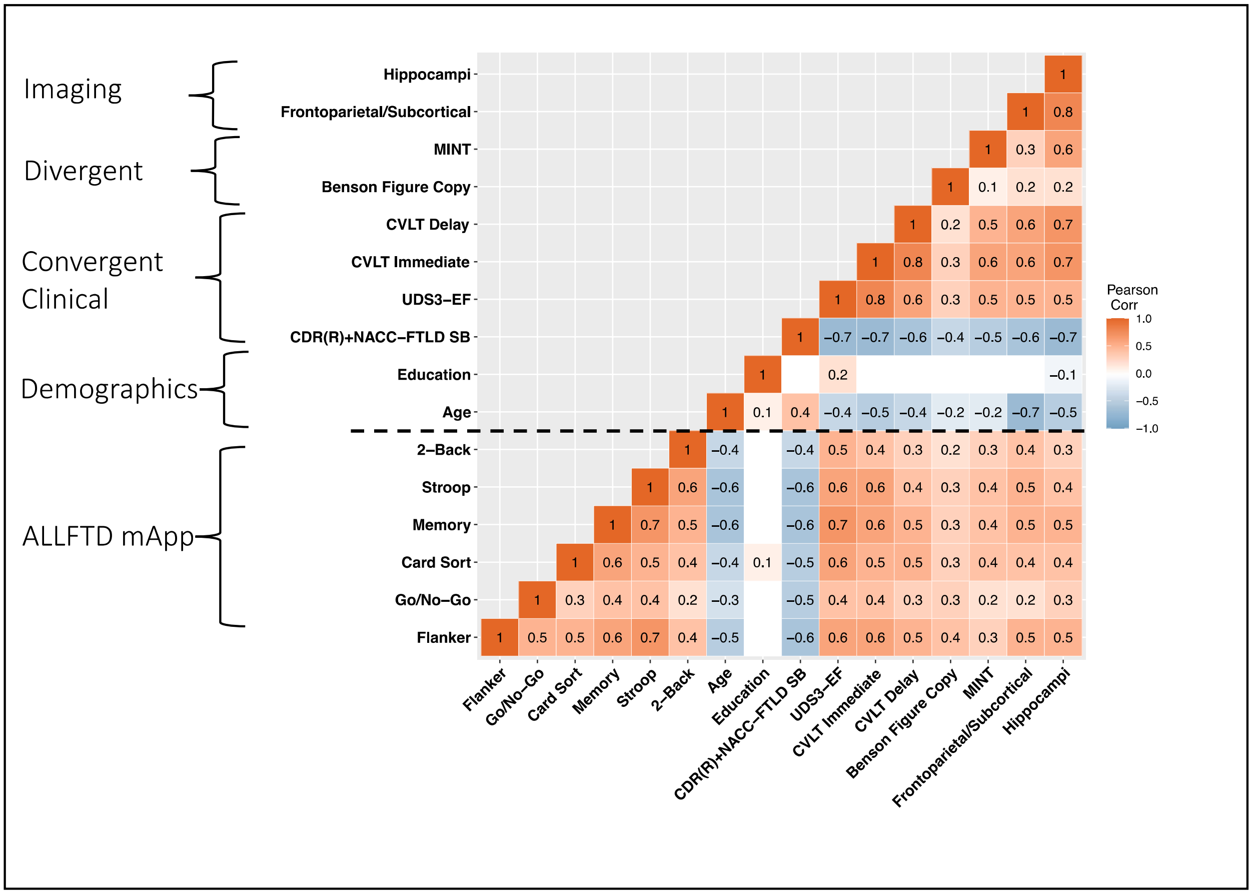

Note: Correlation matrix of in-clinic gold standard measures and ALLFTD mApp test scores. Only statistically significant ($P < 0.05$) correlations are displayed. Below the horizontal dashed line, the associations among app tests, and between app tests and demographics, convergent clinical measures, divergent cognitive tests, and neuroimaging ROIs can be viewed. Most app tests show strong correlations with each other and with age, convergent clinical measures, and brain volume. The measures show weaker associations with divergent measures of visuospatial (Benson) and language (MINT) abilities. The strength of convergent associations between app measures and outcomes is similar to the association between gold-standard neuropsychological scores and these outcomes, which can be viewed by looking across the rows above the horizontal black line.

© 2024 Staffaroni AM et al. *JAMA Network Open*.

eFigure 5. Neural Correlates of Smartphone Cognitive Test Performance

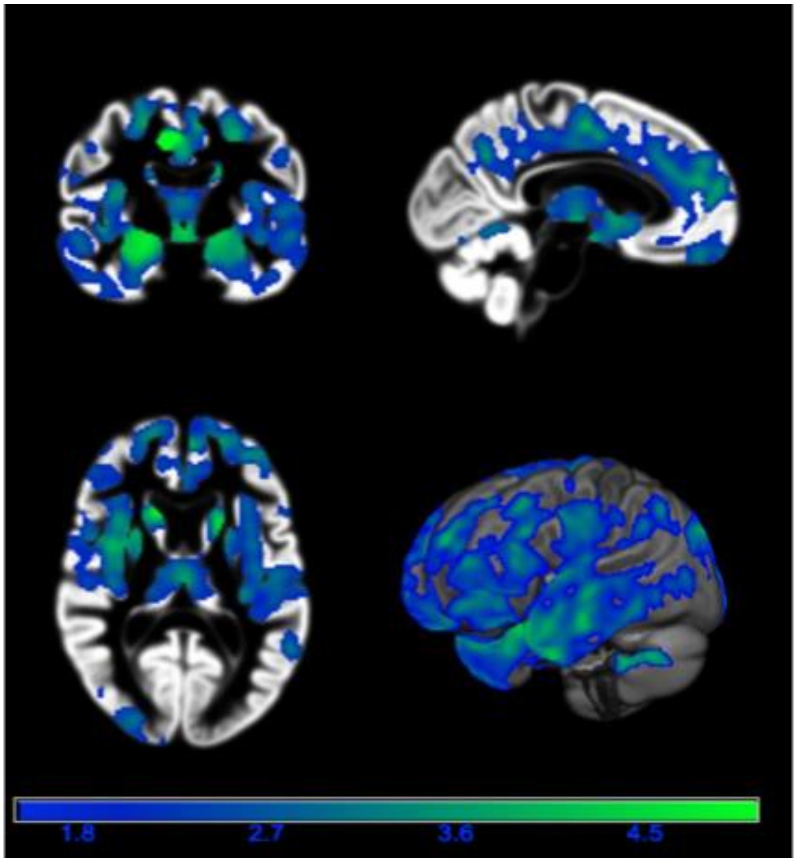

Note. These renderings show voxel-wise gray matter correlates of a composite of five ALLFTD mApp tests (excluding Go/No-Go). T-statistics are presented only in voxels that were statistically significant ($P < 0.05$) after family-wise error correction.

© 2024 Staffaroni AM et al. *JAMA Network Open*.

eReferences

1. Weintraub S, Besser L, Dodge HH, et al. Version 3 of the Alzheimer Disease Centers' Neuropsychological Test Battery in the Uniform Data Set (UDS). *Alzheimer Dis Assoc Disord*. 2018;32(1):10-17. doi:10.1097/WAD.0000000000000223
2. Krueger CE, Bird AC, Growdon ME, Jang JY, Miller BL, Kramer JH. Conflict monitoring in early frontotemporal dementia. *Neurology*. 2009;73(5):349-355. doi:10.1212/WNL.0b013e3181b04b24
3. Kramer JH, Mungas D, Possin KL, et al. NIH EXAMINER: conceptualization and development of an executive function battery. *J Int Neuropsychol Soc*. 2014;20(1):11-19. doi:10.1017/S1355617713001094
4. Staffaroni AM, Bajorek L, Casaletto KB, et al. Assessment of executive function declines in presymptomatic and mildly symptomatic familial frontotemporal dementia: NIH-EXAMINER as a potential clinical trial endpoint. *Alzheimers Dement*. 2020;16(1):11-21. doi:10.1016/j.jalz.2019.01.012
5. Weintraub S, Dikmen SS, Heaton RK, et al. Cognition assessment using the NIH Toolbox. *Neurology*. 2013;80(Issue 11, Supplement 3):S54-S64. doi:10.1212/WNL.0b013e3182872ded
6. Diamond A. Executive Functions. *Annu Rev Psychol*. 2013;64(1):135-168. doi:10.1146/annurev-psych-113011-143750
7. Stroop JR. Studies of interference in serial verbal reactions. *J Exp Psychol*. 1935;18(6):643-662.
8. Rizopoulos D. ltm: An R Package for Latent Variable Modeling and Item Response Analysis. *J Stat Softw*. 2006;17(5 SE-Articles):1-25. doi:10.18637/jss.v017.i05
9. Wolak M, Wolak MM. Package "ICC" Facilitating Estimation of the Intraclass Correlation Coefficient. Published online 2015.
10. Olney NT, Ong E, Goh SYM, et al. Clinical and volumetric changes with increasing functional impairment in familial frontotemporal lobar degeneration. *Alzheimer's and Dementia*. 2020;16(1):49-59. doi:10.1016/j.jalz.2019.08.196
11. Sled JG, Zijdenbos AP, Evans AC. A nonparametric method for automatic correction of intensity nonuniformity in MRI data. *IEEE Trans Med Imaging*. 1998;17(1):87-97. doi:10.1109/42.668698
12. Ashburner J, Friston KJ. Unified segmentation. *Neuroimage*. 2005;26(3):839-851. doi:10.1016/j.neuroimage.2005.02.018
13. Ashburner J, Friston KJ. Diffeomorphic registration using geodesic shooting and Gauss-Newton optimisation. *Neuroimage*. 2011;55(3):954-967. doi:10.1016/j.neuroimage.2010.12.049
14. Desikan RS, Ségonne F, Fischl B, et al. An automated labeling system for subdividing the human cerebral cortex on MRI scans into gyral based regions of interest. *Neuroimage*. 2006;31(3):968-980. doi:10.1016/j.neuroimage.2006.01.021
15. Ashburner J, Friston KJ. Voxel-based morphometry - The methods. *Neuroimage*. 2000;11(6 I):805-821. doi:10.1006/nimg.2000.0582
16. Winkler AM, Ridgway GR, Webster MA, Smith SM, Nichols TE. Permutation inference for the general linear model. *Neuroimage*. 2014;92:381-397. doi:10.1016/j.neuroimage.2014.01.060

© 2024 Staffaroni AM et al. *JAMA Network Open*.
