# Supplementary material for: Reliability and Validity of Smartphone Cognitive Testing for Frontotemporal Lobar Degeneration
Source: JAMA Netw Open. 2024 Apr 1;7(4):e244266. doi: 10.1001/jamanetworkopen.2024.4266 (PMC10985553; doi:10.1001/jamanetworkopen.2024.4266)
Supplement: Supplement 2. — Nonauthor Collaborators [file jamanetwopen-e244266-s002.pdf]

Supplemental Online Content: Nonauthor Collaborators
*First name, last name, and suffix (if applicable) are required and will appear in PubMed.

*Group Name(s): ALLFTD Consortium							
***First Name and Middle Initial(s)**	***Last Name**	***Suffix (eg, Jr, III)**	Academic Degrees	Institution	Location (city, state/province, country)	Role or Contribution, eg, chair, principal investigator	Group (if more than 1 Group listed in the byline) and/or Subgroup (eg, Steering Committee)
Liana	Apostolova		MD	Indiana University	Indianapolis, IN, USA	Site PI	
Brian	Appleby		MD	Case Western Reserve University	Cleveland, OH, USA	Site PI	
Sami	Barmada		MD, PhD	University of Michigan	Ann Arbor, MI, USA	Site PI	
Ece	Bayram		MD, PhD	UCSD	San Diego, CA, USA	Clinician	
Bradley	Boeve		MD	Mayo Clinic, Rochester	Rochester, MN, USA	Study MPI	
Hugo	Botha		MD	Mayo Clinic, Rochester	Rochester, MN, USA	Clinician	
Adam L.	Boxer		MD, PhD	UCSF	San Francisco, CA, USA	Study MPI	
Andrea	Bozoki		MD	University of North Carolina	Chapel Hill, NC, USA	Site PI	
Danielle	Brushaber		BS	Mayo Clinic, Rochester	Rochester, MN, USA	Data Management	
Annie L.	Clark		MS	UCSF	San Francisco, CA, USA	Data Management	
Yann	Cobigo		PhD	UCSF	San Francisco, CA, USA	Data Management	
R. Ryan	Darby		MD	Vanderbilt University	Nashville, TN, USA	Site PI	
Gregg S	Day		MD, MSc	Mayo Clinic Jacksonville	Jacksonville, FL, USA	Clinician	
Sreya	Dhanam		BS	UCSF	San Francisco, CA, USA	Study Coordinator	
Bradford	Dickerson		MD	MGH	Boston, MA, USA	Site PI	
Dennis	Dickson		MD	Mayo Clinic, Jacksonville	Jacksonville, FL, USA	Neuropathology	
Kimiko	Domoto-Reilly		MD	University of Washington	Seattle, WA, USA	Site PI	
Fanny	Elahi		MD, PhD	Icahn School of Medicine at Mount Si	New York, NY, USA	Site PI	
Kelley	Faber		MS	Indiana University (NCRAD)	Indianapolis, IN, USA	Biospecimens	
Anne	Fagan		PhD	Washington University, St. Louis	St. Louis, MO, USA	Biospecimens	
Julie A.	Fields		PhD	Mayo Clinic, Rochester	Rochester, MN, USA	Neuropsychology	
Jamie	Fong		MS	UCSF	San Francisco, CA, USA	Genetic Counselor	
Tatiana	Foroud		PhD	Indiana University (NCRAD)	Indianapolis, IN, USA	Biospecimens	
Leah K.	Forsberg		PhD	Mayo Clinic, Rochester	Rochester, MN, USA	Executive	
Douglas R.	Galasko		MD	UCSD	San Diego, CA, USA	Site PI	
Ralitza	Gavrilova		MD	Mayo Clinic, Rochester	Rochester, MN, USA	Genetic Counselor	
Tania	Gendron		PhD	Mayo Clinic Jacksonville	Jacksonville, FL, USA	Biospecimens	
Daniel	Geschwind		MD, PhD	UCLA	Los Angeles, CA, USA	Genetics	
Nupur	Ghoshal		MD	Washington University, St. Louis	St. Louis, MO, USA	Site PI	

Supplemental Online Content: Nonauthor Collaborators
*First name, last name, and suffix (if applicable) are required and will appear in PubMed.

***First Name and Middle Initial(s)**	***Last Name**	***Suffix (eg, Jr, III)**	Academic Degrees	Institution	Location (city, state/province, country)	Role or Contribution, eg, chair, principal investigator	Group (if more than 1 Group listed in the byline) and/or Subgroup (eg, Steering Committee)
Jill	Goldman		MS, MPhil	Columbia University	New York, NY, USA	Genetic Counselor	
Neill	Graff-Radford		MBBCh, FR	Mayo Clinic Jacksonville	Jacksonville, FL, USA	Site PI	
Jonathan	Graff-Radford		MD	Mayo Clinic, Rochester	Rochester, MN, USA	Clinician	
Ian M.	Grant		MD	Northwestern University	Chicago, IL, USA	Site PI	
Murray	Grossman		MD, EdD	University of Pennsylvania	Philadelphia, PA, USA	Site PI	
Matthew	Hall		MS	UCSF	San Francisco, CA, USA	Genetic Counselor	
Chadwick M.	Hales		MD, PhD	Emory	Atlanta, GA, USA	Site PI	
Hilary W.	Heuer		PhD	UCSF	San Francisco, CA, USA	Executive	
Lawrence S.	Honig		MD	Columbia University	New York, NY, USA	Site PI	
Ging-Yuek (Robin)	Hsiung		MD	University of British Columbia	Vancouver, British Columb	Site PI	
Eric	Huang		PhD	UCSF	San Francisco, CA, USA	Biospecimens	
Edward D.	Huey		MD	Brown University	Providence, RI, USA	Site PI	
David	Irwin		MD	University of Pennsylvania	Philadelphia, PA, USA	Site PI	
Noah	Johnson		BA	Mayo Clinic, Rochester	Rochester, MN, USA	Data Management	
David T.	Jones		MD	Mayo Clinic, Rochester	Rochester, MN, USA	Clinician	
Kejal	Kantarci		MD	Mayo Clinic, Rochester	Rochester, MN, USA	Imaging	
David	Knopman		MD	Mayo Clinic, Rochester	Rochester, MN, USA	Site PI	
Tyler	Kolander		BA	Mayo Clinic, Rochester	Rochester, MN, USA	Data Management	
John	Kornak		PhD	UCSF	San Francisco, CA, USA	Statistics	
Walter	Kremers		PhD	Mayo Clinic, Rochester	Rochester, MN, USA	Statistics	
Justin	Kwan		MD	National Institutes of Health	Bethesda, MD, USA	Site PI	
Argentina	Lario Lago		PhD	UCSF	San Francisco, CA, USA	Biospecimens	
Maria	Lapid		MD	Mayo Clinic, Rochester	Rochester, MN, USA	Clinician	
Shannon B.	Lavigne		PhD	UT Health San Antonio	San Antonio, TX, USA	Neuropsychology	
Suzee	Lee		MD	UCSF	San Francisco, CA, USA	Clinician	
Gabriel C.	Léger		MD	UCSD	San Diego, CA, USA	Site PI	
Irene	Litvan		MD, MSc	UCSD	San Diego, CA, USA	Site PI	
Peter	Ljubenkov		MD	UCSF	San Francisco, CA, USA	Clinician	
Diane	Lucente		MS	MGH	Boston, MA, USA	Genetic Counselor	
Ian R.	Mackenzie		MD	University of British Columbia	Vancouver, British Columb	Site PI	

Supplemental Online Content: Nonauthor Collaborators
*First name, last name, and suffix (if applicable) are required and will appear in PubMed.

***First Name and Middle Initial(s)**	***Last Name**	***Suffix (eg, Jr, III)**	Academic Degrees	Institution	Location (city, state/province, country)	Role or Contribution, eg, chair, principal investigator	Group (if more than 1 Group listed in the byline) and/or Subgroup (eg, Steering Committee)
Masood	Manoochehri		BA	Columbia University	New York, NY, USA	Study Coordinator	
Joseph C.	Masdeu		MD, PhD	Houston Methodist	Houston, TX, USA	Site PI	
Lauren	Massimo		PhD	University of Pennsylvania	Philadelphia, PA, USA	Clinician	
Scott	McGinnis		MD	MGH	Boston, MA, USA	Clinician	
Corey T.	McMillan		PhD	University of Pennsylvania	Philadelphia, PA, USA	Clinician	
Mario F.	Mendez		MD, PhD	UCLA	Los Angeles, CA, USA	Site PI	
Carly	Mester		BA	Mayo Clinic, Rochester	Rochester, MN, USA	Data Management	
Joie	Molden		PhD	University of Colorado	Aurora, CO, USA	Neuropsychology	
Toji	Miyagawa		MD, PhD	Mayo Clinic, Rochester	Rochester, MN, USA	Clinician	
Georges	Naasan		MD	Icahn School of Medicine at Mount Si	New York, NY, USA	Site PI	
Chiadi	Onyike		MBBS, MH:	Johns Hopkins University	Baltimore, MD, USA	Site PI	
Alexander	Pantelyat		MD	Johns Hopkins University	Baltimore, MD, USA	Clinician	
Emily	Paolillo		PhD	UCSF	San Francisco, CA, USA	Remote App	
Belen	Pascual		PhD	Houston Methodist	Houston, TX, USA	Site PI	
Henry	Paulson		MD, PhD	University of Michigan	Ann Arbor, MI, USA	Clinician	
Leonard	Petrucelli		PhD	Mayo Clinic Jacksonville	Jacksonville, FL, USA	Biospecimens	
Peter	Pressman		MD	University of Colorado	Aurora, CO, USA	Site PI	
Rosa	Rademakers		PhD	University of Antwerp	Antwerpen, Belgium	Genetics	
Vijay	Ramanan		MD, PhD	Mayo Clinic, Rochester	Rochester, MN, USA	Clinician	
Eliana Marisa	Ramos		PhD	UCLA	Los Angeles, CA, USA	Genetics	
Katherine P.	Rankin		PhD	UCSF	San Francisco, CA, USA	Neuropsychology	
Meghana	Rao		BS, MPH	Mayo Clinic, Rochester	Rochester, MN, USA	Data Management	
Katya	Rascovsky		PhD	University of Pennsylvania	Philadelphia, PA, USA	Neuropsychology	
Kristoffer W.	Rhoads		PhD	University of Washington	Seattle, WA, USA	Neuropsychology	
Jessica	Rexach		MD, PhD	UCLA	Los Angeles, CA, USA	Genetics	
Aaron	Ritter		MD	Cleveland Clinic Las Vegas	Las Vegas, NV, USA	Site PI	
Erik D.	Roberson		MD, PhD	University of Alabama at Birmingham	Birmingham, AL, USA	Site PI	
Emily	Rogalski		PhD	Northwestern University	Chicago, IL, USA	Site PI	
Julio C.	Rojas		MD, PhD	UCSF	San Francisco, CA, USA	Clinician	
Howard J.	Rosen		MD	UCSF	San Francisco, CA, USA	Study MPI	

Supplemental Online Content: Nonauthor Collaborators
*First name, last name, and suffix (if applicable) are required and will appear in PubMed.

***First Name and Middle Initial(s)**	***Last Name**	***Suffix (eg, Jr, III)**	Academic Degrees	Institution	Location (city, state/province, country)	Role or Contribution, eg, chair, principal investigator	Group (if more than 1 Group listed in the byline) and/or Subgroup (eg, Steering Committee)
Rodolfo	Savica		MD, PhD	Mayo Clinic, Rochester	Rochester, MN, USA	Clinician	
William	Seeley		MD	UCSF	San Francisco, CA, USA	Neuropathology	
Allison	Snyder		MD	National Institutes of Health	Bethesda, MD, USA	Site PI	
Adam M.	Staffaroni		PhD	UCSF	San Francisco, CA, USA	Neuropsychology	
Anne C.	Sullivan		PhD	UT Health San Antonio	San Antonio, TX, USA	Site PI	
Jeremy M.	Syrjanen		MS	Mayo Clinic, Rochester	Rochester, MN, USA	Data Management	
M. Carmela	Tartaglia		MD	University of Toronto	Toronto, Ontario, Canada	Site PI	
Jack	Taylor		MA	UCSF	San Francisco, CA, USA	Remote App	
Philip W.	Tipton		MD	Mayo Clinic Jacksonville	Jacksonville, FL, USA	Clinician	
Marijne	Vandebergh		PhD	University of Antwerp	Antwerpen, Belgium	Genetics	
Arthur	Toga		PhD	Keck School of Medicine of University	Los Angeles, CA, USA	Imaging	
Lawren	VandeVrede		MD, PhD	UCSF	San Francisco, CA, USA	Clinician	
Sandra	Weintraub		PhD	Northwestern University	Chicago, IL, USA	Neuropsychology	
Dylan	Wint		MD	Cleveland Clinic Las Vegas	Las Vegas, NV, USA	Site PI	
Amy B.	Wise		BA	UCSF	San Francisco, CA, USA	Study Coordinator	
Amy	Wolf		BS	UCSF	San Francisco, CA, USA	Data Management	
Bonnie	Wong		PhD	MGH	Boston, MA, USA,	Neuropsychology	
Zbigniew K.	Wszolek		MD	Mayo Clinic, Jacksonville	Jacksonville, FL, USA	Clinician	
Jennifer	Yokoyoma		PhD	UCSF	San Francisco, CA, USA	Genetics	
