# Supplementary material for: Reliability and Validity of Smartphone Cognitive Testing for Frontotemporal Lobar Degeneration
Source: JAMA Netw Open. 2024 Apr 1;7(4):e244266. doi: 10.1001/jamanetworkopen.2024.4266 (PMC10985553; doi:10.1001/jamanetworkopen.2024.4266)
Supplement: Supplement 3. — Data Sharing Statement [file jamanetwopen-e244266-s003.pdf]

Data Sharing Statement

Staffaroni. Reliability and Validity of Smartphone Cognitive Testing for Frontotemporal Lobar Degeneration. *JAMA Netw Open*. Published April 01, 2024.
doi:10.1001/jamanetworkopen.2024.4266

Data
Data available: Yes
Data types: Deidentified participant data, Data dictionary
How to access data: Qualified researchers can request data through the ALLFTD consortium website (https://www.allftd.org/data).
When available: With publication

Supporting Documents
Document types: None

Additional Information
Who can access the data: In accordance with ALLFTD data sharing policies (https://www.allftd.org/policies), data will be made available to qualified researchers whose proposed use of the data has been approved.
Types of analyses: Data requests will be reviewed for scientific merit and will consider potential overlap with ongoing analyses.
Mechanisms of data availability: Data will be made available after approval of a proposal
